# Myosin Sequestration Regulates Sarcomere Function, Cardiomyocyte Energetics, and Metabolism, Informing the Pathogenesis of Hypertrophic Cardiomyopathy

**DOI:** 10.1161/CIRCULATIONAHA.119.042339

**Published:** 2020-01-27

**Authors:** Christopher N. Toepfer, Amanda C. Garfinkel, Gabriela Venturini, Hiroko Wakimoto, Giuliana Repetti, Lorenzo Alamo, Arun Sharma, Radhika Agarwal, Jourdan F. Ewoldt, Paige Cloonan, Justin Letendre, Mingyue Lun, Iacopo Olivotto, Steve Colan, Euan Ashley, Daniel Jacoby, Michelle Michels, Charles S. Redwood, Hugh C. Watkins, Sharlene M. Day, James F. Staples, Raúl Padrón, Anant Chopra, Carolyn Y. Ho, Christopher S. Chen, Alexandre C. Pereira, Jonathan G. Seidman, Christine E. Seidman

**Affiliations:** 1 Department of Genetics, Harvard Medical School, Boston, MA (C.N.T., A.C.G., G.V., H.W., G.R., A.S., R.A., A.C.P., J.G.S., C.E.S.).; 2 Cardiovascular Medicine, Radcliffe Department of Medicine (C.N.T., C.S.R., H.C.W.), University of Oxford, UK.; 3 Wellcome Centre for Human Genetics (C.N.T., H.C.W.), University of Oxford, UK.; 4 Laboratory of Genetics and Molecular Cardiology, Heart Institute (InCor)-University of São Paulo Medical School, Brazil (G.V., A.C.P.).; 5 Centro de Biología Estructural, Instituto Venezolano de Investigaciones Cientifìcas (IVIC), Caracas (L.A., R.P.).; 6 Department of Biomedical Engineering, Boston University, MA (J.F.E., P.C., J.L., A.C., C.S.C.).; 7 Department of Medicine, Division of Genetics (M.L.), Brigham and Women’s Hospital, Boston, MA.; 8 Cardiovascular Division (C.Y.H., C.E.S.), Brigham and Women’s Hospital, Boston, MA.; 9 Cardiomyopathy Unit and Genetic Unit, Careggi University Hospital, Florence, Italy (I.O.).; 10 Department of Cardiology, Boston Children’s Hospital, MA (S.C.).; 11 Center for Inherited Cardiovascular Disease, Stanford University, CA (E.A.).; 12 Department of Internal Medicine, Section of Cardiovascular Diseases, Yale School of Medicine, New Haven, CT (D.J.).; 13 Department of Cardiology, Thorax Center, Erasmus MC, Rotterdam, The Netherlands (M.M.).; 14 Department of Internal Medicine, University of Michigan, Ann Arbor (S.M.D.).; 15 Department of Biology, University of Western Ontario, London, Canada (J.F.S.).; 16 Division of Cell Biology and Imaging, Department of Radiology, University of Massachusetts Medical School, Worcester (R.P.).; 17 Howard Hughes Medical Institute, Chevy Chase, MD (C.E.S.).

**Keywords:** cardiomyopathy, hypertrophic, cardiovascular physiological phenomena, myosins, sarcomeres

## Abstract

**Background:**

Hypertrophic cardiomyopathy (HCM) is caused by pathogenic variants in sarcomere protein genes that evoke hypercontractility, poor relaxation, and increased energy consumption by the heart and increased patient risks for arrhythmias and heart failure. Recent studies show that pathogenic missense variants in myosin, the molecular motor of the sarcomere, are clustered in residues that participate in dynamic conformational states of sarcomere proteins. We hypothesized that these conformations are essential to adapt contractile output for energy conservation and that pathophysiology of HCM results from destabilization of these conformations.

**Methods:**

We assayed myosin ATP binding to define the proportion of myosins in the super relaxed state (SRX) conformation or the disordered relaxed state (DRX) conformation in healthy rodent and human hearts, at baseline and in response to reduced hemodynamic demands of hibernation or pathogenic HCM variants. To determine the relationships between myosin conformations, sarcomere function, and cell biology, we assessed contractility, relaxation, and cardiomyocyte morphology and metabolism, with and without an allosteric modulator of myosin ATPase activity. We then tested whether the positions of myosin variants of unknown clinical significance that were identified in patients with HCM, predicted functional consequences and associations with heart failure and arrhythmias.

**Results:**

Myosins undergo physiological shifts between the SRX conformation that maximizes energy conservation and the DRX conformation that enables cross-bridge formation with greater ATP consumption. Systemic hemodynamic requirements, pharmacological modulators of myosin, and pathogenic myosin missense mutations influenced the proportions of these conformations. Hibernation increased the proportion of myosins in the SRX conformation, whereas pathogenic variants destabilized these and increased the proportion of myosins in the DRX conformation, which enhanced cardiomyocyte contractility, but impaired relaxation and evoked hypertrophic remodeling with increased energetic stress. Using structural locations to stratify variants of unknown clinical significance, we showed that the variants that destabilized myosin conformations were associated with higher rates of heart failure and arrhythmias in patients with HCM.

**Conclusions:**

Myosin conformations establish work-energy equipoise that is essential for life-long cellular homeostasis and heart function. Destabilization of myosin energy-conserving states promotes contractile abnormalities, morphological and metabolic remodeling, and adverse clinical outcomes in patients with HCM. Therapeutic restabilization corrects cellular contractile and metabolic phenotypes and may limit these adverse clinical outcomes in patients with HCM.

## Abstract 2

Supplemental Digital Content is available in the text.

**Clinical Perspective**

**What Is New?**

- The proportion of myosin molecules in 2 conformations (super relaxed state; SRX and disordered relaxed state; DRX) provide a fundamental mechanism for enabling efficient myocardial performance and conservation of energy.
- Hypertrophic cardiomyopathy (HCM) mutations that disrupt the physiological balance myosins in the SRX and DRX conformations alter cardiomyocyte contraction, relaxation, and metabolism, and convey increased risks for heart failure and atrial fibrillation.
- A small molecule can restore the physiological balance of myosins in the SRX and DRX conformations and disordered relaxed state of myosin molecule and improve functional, energetic, and cellular abnormalities that occur in patients with HCM.

**What Are the Clinical Implications?**

- Normal relaxation by cardiomyocytes is profoundly important for life-long heart function, and the disruption of this property increases risks for adverse clinical outcomes.
- The identity of amino acids that participate in conformational interactions during cardiac relaxation provides a new strategy to better classify HCM variants, including those of unknown significance, to define patients with HCM and high risks for adverse outcomes.
- A small molecule that normalizes the fundamental abnormalities caused by HCM mutations provides a therapeutic opportunity to limit the development of hypertrophy, diastolic dysfunction, atrial fibrillation, and heart failure in patients with HCM.

Muscle cells must balance work demands with energetic costs, processes that are particularly critical for cardiomyocytes that continuously function in heart muscle. Cardiac work must also accommodate vast changes in systemic hemodynamic parameters that are high during intense physical exercise but low during the deepest phases of sleep when energy can be conserved. Although overall cardiac performance is modulated by multiple, integrated signals emanating from many organ systems, fundamentally these converge at the sarcomere, the essential unit of contraction in cardiomyocytes and other muscle cells. In the heart, sarcomere power is regulated by changes in beat rate, sarcomere length, denoted as length-dependent activation,^1,2^ intracellular calcium flux,^3^ and phosphorylation of titin, troponin I, myosin-binding protein C, and myosin regulatory chain.^4–9^ When greater sarcomere power is needed, these processes augment the numbers of myosin molecules available for cross-bridge formation with the thin filament and increase ATP consumption.

Myosin is the enzymatic molecular motor of the sarcomere that hydrolyzes ATP to propel cyclical interactions with thin filament actin. Within sarcomeres, only 10% of myosin molecules are estimated to be involved in sarcomere force production,^10^ an observation that implies a mechanism(s) whereby unneeded myosins are prevented from forming inappropriate cross bridges causing unnecessary energy expenditure. Biochemical analyses^11^ and recent structural data^12,13^ imply that dynamic conformational changes between paired myosin molecules provide a structural mechanism for this energy-conserving state. During relaxation, head domains of paired myosin molecules appear to engage via an interacting-heads motif (IHM), and assume either a super relaxed state (SRX) conformation in which both ATP-binding domains are sterically inhibited and unable to bind actin, or a disordered state (DRX) conformation in which only one myosin head is available to hydrolyze ATP and interact with actin.^12^ This model predicts that the proportion of myosins in these conformations would influence both contractility and energy usage and provide a mechanism to balance work and metabolic costs that enables life-long cardiac function.

*MYH7* and *MYBPC3*, which respectively encode the predominant adult cardiac myosin isoform and myosin-binding protein C, harbor the majority of pathogenic variants that cause hypertrophic cardiomyopathy (HCM),^14^ a primary myocardial disorder characterized by hyperdynamic contraction, poor relaxation, and increased energy consumption. Most HCM variants in *MYBPC3* encode truncations that increase myosin contractility,^15^ concurrent with a shift in the proportion of myosins in DRX.^15–17^ By contrast, all HCM variants in *MYH7* encode missense residues and the cellular effects of these subtle changes on myosin SRX and DRX conformations are largely unknown. Indeed, previous biophysical and biochemical analyses of isolated single myosin head fragments with HCM variants do not consistently demonstrate gain of function,^14,18–24^ as might be predicted by a conformational shift toward DRX.

HCM is clinically diagnosed by imaging studies that demonstrate increased ventricular wall thickness, or hypertrophy, a morphological phenotype that is presumed to be caused by enlarged cardiomyocytes and increased amounts of myocardial fibrosis. HCM often causes arrhythmias, most often atrial fibrillation, and, in some patients, heart failure and premature death. Recent analyses of >4500 patients with HCM showed that heart failure and arrhythmic risks were tiered according to sarcomere protein genotype: highest in patients with pathogenic or likely pathogenic variants, intermediate among patients with variants of unknown clinical significance (VUS), and lowest in patients without sarcomere variants.^14^ These epidemiological observations infer that clinical outcomes may reflect different biophysical properties of HCM variants and their effects on the cardiomyocyte biology.

Here we probed the unknown physiological effects of myosin DRX and SRX conformations on cardiomyocyte biology. We tested whether the evolutionarily conserved IHM^25^ provided an adaptable mechanism to promote myosin in the SRX or DRX conformations, by studying hibernating mammalian hearts in different stages of torpor or arousal. We contrasted these analyses with studies of human cardiomyocytes with normal myosin sequences or with pathogenic variants that disrupted IHM residues. We determined the effects on myosin SRX and DRX conformations, and cardiomyocyte contractility, sarcomere content, cell size, and cell metabolism. Using a myosin allosteric modulator, MYK-461, we assayed the direct cellular effects of altering the balance of myosin conformational states. Lastly, we extended experimentation to address whether changes in biophysical and cellular properties associated with myosin VUS correlated with clinical adverse outcomes in patients with HCM. Together our data provide deeper insights into the importance of regulating myosin conformations in health and disease.

## Methods

The data that support the findings of this study are available within the article and the online-only Data Supplement. Requests for reagents and anonymized human data from qualified researchers trained in human subject confidentiality protocols may be sent to the corresponding authors.

We studied HCM mutations in myosin using mouse models, isogenic human cardiomyocytes, and heart tissues with different background genotypes. Mouse models express mutations in *Myh6*, whereas isogenic cardiomyocytes derived from induced pluripotent stem cells (iPSC-CMs) express mutations in *MYH7*. Comparative assays of mouse and human cardiomyocytes were used to determine if functional differences observed in mutations were influenced by these species-specific myosin isoforms. Human cardiomyocytes were derived from a parental induced pluripotent stem cell line that carried a green-fluorescent protein tag on one titin allele (denoted GFP-tagged iPSC-CMs).^26,27^ HCM variants were engineered into the endogenous *MYH7* gene in this line to compare mutational effects within an isogenic background. Mutant iPSC-CMs were used to study live cell contractility, energetics, and metabolism. Parallel analyses in isolated cardiomyocytes from mouse HCM models and human HCM heart tissues were used to validate that iPSC-CM findings also occurred in vivo.

### Rodent Studies

All animal experiments were performed in accordance with protocols that were reviewed and approved by institutional boards in accordance with the Animal Care and Use Committee of the University of Western Ontario and Harvard Medical School. Ventricular heart tissues were snap frozen in liquid N_2_ immediately on excision from 13-lined ground squirrel (*Ictidomys tridecemlineatus*) during torpor, interbout euthermia, and arousal, as previously described.^28^ Cardiomyocytes and heart tissues were isolated from mice carrying human HCM mutations in the endogenous *Myh6* gene,^29,30^ as previously described.^11,15^ Contraction and relaxation measurements of isolated cardiomyocytes from 3 mice with identical genotype were performed as described previously.^15^

### Human Studies

Human subjects participating in the Sarcomeric Human Cardiomyopathy Registry (SHaRe) were recruited after obtaining written signed consent using protocols that were reviewed and approved by each institution of the participating SHaRe investigators, as previously described.^14^ The races and ethnicities of SHaRe participants studied here (Table I in the online-only Data Supplement) and information on left ventricular (LV) myocardial tissue samples (Table II in the online-only Data Supplement) are provided in the Extended Methods in the online-only Data Supplement.

All participants clinically diagnosed with HCM based on the finding of isolated and unexplained LV hypertrophy (maximal LV wall thickness ≥ 13 mm or *Z*-score for pediatric patients). Additional inclusion criteria required ≥1 clinic visit at a SHaRe site since 1960, ≥1 echocardiographic assessments of LV wall thickness, and available genetic test data on 8 sarcomere HCM genes: myosin-binding protein C (*MYBPC3*), β-myosin heavy chain (*MYH7*), cardiac troponin T (*TNNT2*), cardiac troponin I (*TNNI3*), α-tropomyosin (*TPM1*), myosin essential and regulatory light chains (*MYL3*, *MYL2*, respectively), and actin (*ACTC*). Sarcomere variants in these genes were reviewed by a subgroup of SHaRe investigators to standardize classification,^14^ and patients were designated as SARC+ (≥1 pathogenic or likely pathogenic variant in sarcomere genes), SARC VUS (in one of the sarcomere genes listed above), or SARC– (no pathogenic or likely variant in sarcomere genes). Patients with pathogenic or likely pathogenic variants in other genes implicated in LV hypertrophy were excluded.

Among 3695 patients meeting these criteria we selected all patients (n=2281) assigned as SARC+ or SARC VUS on the basis of *MYH7*, *MYL2*, or *MYL3* genotypes, because these genes participate in the IHM.^12^ SARC VUS genotypes (n=133) were reclassified on the basis of whether the variant altered an amino acid residue involved in the IHM (VUS IHM+) or not (VUS IHM–) using the previously described quasi-atomic IHM model (Research Collaboratory for Structural Bioinformatics, Protein Data Bank entry 5TBY and supplementary file 2 in reference ^12^).

We compared the incidence of adverse outcomes by using Kaplan-Meier analyses based on genotypes. Time to first occurrence of atrial fibrillation, a composite score of heart failure–related outcomes (time to first occurrence of cardiac transplantation, LV assist device implantation, LV ejection fraction <35%, or New York Heart Association class III/IV symptoms), and an overall composite including these metrics, and all-cause death, as well, were analyzed. These adverse events were analyzed from birth to assess the lifetime cumulative incidence of clinical outcomes. Patients who did not have the outcome under analysis were censored at the time point of their last recorded visit in SHaRe. Patients without data (occurrence or timing of an outcome) were excluded from analysis of that outcome.

LV heart samples were obtained from a subset of SHaRe participants undergoing procedures for clinical indications at Brigham and Women’s Hospital and the University of Michigan Medical Center. Tissues were snap frozen in liquid N_2_ immediately on excision and used in studies detailed below.

### Derivation of Human iPSC-CMs With HCM Variants

Heterozygous HCM variants were introduced into a *MYH7* allele in human isogenic iPSCs carrying GFP-tagged titin and differentiated into cardiomyocytes (iPSC-CMs) as described.^26,27,31^ iPSC-CMs with heterozygous HCM missense variants were studied on day 30 after differentiation, at which time myosin isoform expression was assessed (Table III in the online-only Data Supplement).

### Mant-ATP Assays and Analyses

Mant-ATP assays were performed on 3 samples from mice or human LV and iPSC-CMs (day 30 differentiation) by using a published protocol.^15^ Monolayers of iPSC-CMs were scraped from 6 culture wells or isolated tissues were pinned onto the experimental flow chamber and incubated for 30 minutes (room temperature) in permeabilization buffer. Chambers were then flushed with glycerin solution to allow storage (–20°C for 2 days) or immediate use. Three regions of the chamber were sampled for fluorescence decay analyses as described.^15,17^

### Morphological and Functional Analyses of Day 30 iPSC-CMs

Contractile measures of iPSC-CMs were performed on 3 individual differentiations by using SarcTrack as described.^26,27^

Cell sizes were assessed after incubation with media containing 5 µg/mL wheat germ agglutinin, Alexa Fluor 568 (ThermoFisher Scientific) for 10 minutes at 37°C. After washing, samples were imaged (Nikon Ti Eclipse epifluorescence microscope) and ≥10 images acquired from 10 regions for analyses (MATLAB program; The MathWorks) that quantified pixel intensity units between fluorescent cells and the dark background.

Mitochondrial content was assessed in detached iPSC-CMs, incubated with 250 nmol/L Mitotracker Deep Red (ThermoFisher Scientific; 30 minutes, 37°C, 5% CO_2_). After washing, mitochondria were quantified by using BD FACS Canto II (Becton Dickinson) to assess ≥10 000 events per sample, and data were analyzed using Becton Dickinson FACS Diva. Readings for mean fluorescence intensity were recorded in arbitrary units.

Sarcomere content was assessed in single GFP-tagged iPSC-CMs plated on fibronectin-coated rectangular micro-patterned substrate and polyacrylamide gels, and processed by a custom routine in ImageJ (National Institutes of Health) and MATLAB (Mathworks), as described previously.^32,33^ The spectra of 2-dimenstional Fourier transformed images were converted to a one-dimensional representation, normalized and fitted to a function composed with an aperiodic component (to account for irregular cellular structures) and a periodic component representing regularly spaced sarcomeres. The normalized area under the first nonzero peak of the periodic component was taken as a measure of the regularity of the sarcomere structure.

### Extracellular Flux and Metabolic Analyses of Day 30 iPSC-CMs

Extracellular flux analyses were performed on iPSC-CMs (8×10^4^ cells) seeded on Geltrex-coated XF96 plates, with or without preincubation (24 hours) with MYK-461 (0.5 μmol/L, 1 μmol/L, or vehicle) or 2-deoxy-d-glucose (5 mmol/L or vehicle). Cellular oxygen consumption rate (OCR) and extracellular acidification rate were measured using a Cell Mito Stress Kit and a Seahorse XF96 Analyzer (Agilent Technologies) according to the manufacturer’s instructions.

### Statistical Analyses

Single comparisons were analyzed by using the Student *t* test, with significance defined as *P*<0.05. Multiple comparisons between genotypes were analyzed using 1-way ANOVA with post hoc Bonferroni corrections for multiple comparisons, with significance defined as *P*<0.05. Where multiple comparisons also used MYK-461 treatment, we applied 2-way ANOVA with post hoc Bonferroni corrections for multiple comparisons and assigned significance for *P*<0.05. SHaRe data were analyzed as previously described,^14^ using Kaplan-Meier analyses to assess differences by genotype with Cox proportional hazard modeling that controlled for age at diagnosis and sex.

Additional details are provided in the online-only Data Supplement.

## Results

**Figure 1.**
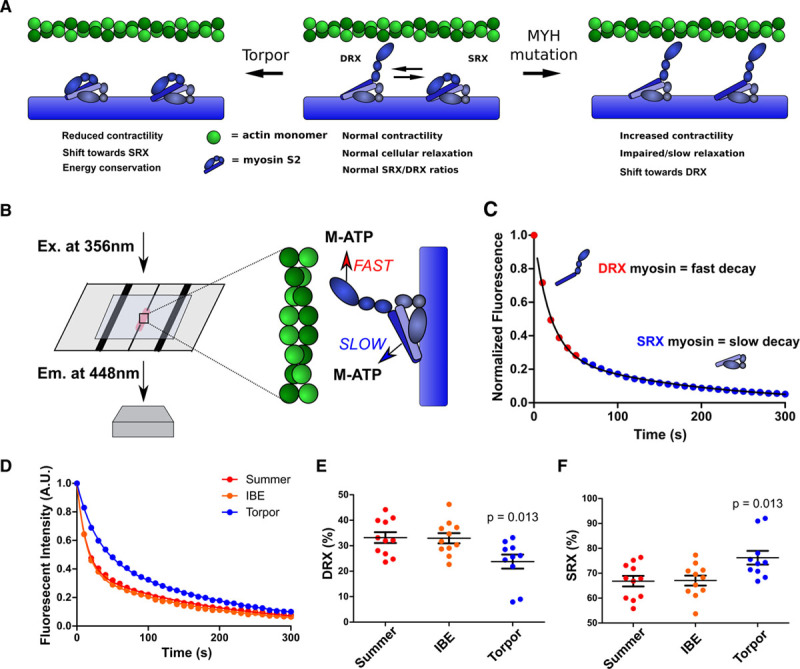
**Mant-ATP assays assess physiological and pathological changes in cardiac myosin conformations.** **A**, Depiction of the predicted myosin SRX and DRX conformations, indicating the dynamic effects of the interacting heads motif, in torpor (**Left**), normal cardiac function, and in hypertrophic cardiomyopathy (**Right**). **B**, Schematics depicting Mant-ATP (M-ATP) infused into a flow chamber containing permeabilized cardiac tissue. A subsequent chase of dark ATP results in fast and slow fluorescence decay (detected at 448 nm using a 40× objective) from DRX and SRX conformations of myosins, respectively. **C**, Representative Mant-ATP fluorescent chase experiment in wild-type tissues, described by a double-exponential decay. **D**, Mant-ATP assays of myocardium from ground squirrel hearts, obtained during summer arousal, interbout euthermia (IBE), and torpor. **E** and **F**, Proportions of myosin heads in the DRX conformation (**E**) with higher rates of ATP cycling and in SRX conformation (**F**) with slower ATP cycling. Data were obtained from studies of 3 hearts in each physiological state, with 3 to 4 samples studied per heart and are plotted as mean±SEM, with significance tested in comparison with summer (arousal), by 1-way ANOVA with Bonferroni correction with a significance cutoff at *P*<0.05. A.U. indicates arbitrary units; DRX, disordered relaxed state conformation of myosin molecule; Em., emission; Ex., excitation; and SRX, super relaxed state conformation of myosin molecule.

Myosin heads that are in the SRX conformation cycle ATP slowly, whereas free myosin heads in the DRX conformation have 5-fold^11^ faster ATP turnover (Figure 1A). With the use of a fluorescent ATP analogue, Mant-ATP, chased with nonfluorescent ATP (Figure 1B), the abundance of myosins in SRX and DRX conformations (Figure 1C) can be deconvoluted from the double-exponential fluorescent decay pattern.^11^ Fast decay reflects myosins in the DRX conformation; slow decay reflects myosins in the SRX conformation.

Using this assay, we previously defined the proportion of myosins in DRX (40%) and SRX (60%) conformations in healthy mouse hearts,^15^ which agrees with a predicted SRX/DRX ratio of 1.5 in relaxed muscle.^12^ To determine if the overall hemodynamic requirements and metabolic state of cardiac physiology correlated with shifts in SRX/DRX ratios, we studied the 13-lined ground squirrel (*I* *tridecemlineatus*), a mammalian hibernator that spends winter in extended bouts of torpor with low core body temperature (5°C), markedly slow heart, respiratory, and metabolic rates (3% basal levels), punctuated by spontaneous arousals to periods of interbout euthermia.^34^ Heart rates (beats/minute, bpm) accurately predict these states and range from ≈340 bpm during arousal, 311 bpm for interbout euthermia, to ≈6 bpm for torpor. Using this model to reflect the extremes in systemic physiological activity, we conducted Mant-ATP assays of hearts obtained during each state (Figure 1D through 1F) and found comparable proportions of myosins in DRX (35%) and SRX (65%) conformations during summertime arousal and interbout euthermia. By contrast, hearts obtained during torpor had increased proportions of the SRX (75%) conformation, suggesting that shifts in the balance of DRX and SRX conformations provided ATP conservation during low needs for cardiac output and limited supplies of energy substrates. Because the IHM is conserved across vertebrate and invertebrate evolution,^25^ we suggest that this mechanism broadly contributes to the efficiency of mammalian muscles.^35^ These observations also imply deleterious effects from chronic distortion of physiological proportions of DRX and SRX conformations.

### HCM Myosin Missense Variants Destabilize the IHM

**Figure 2.**
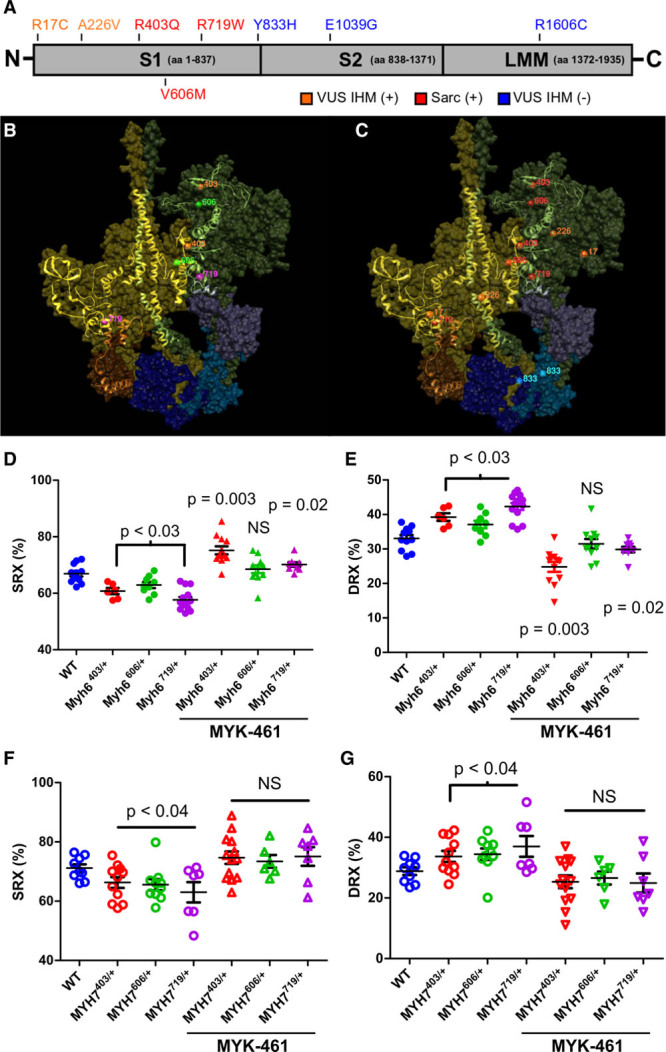
**Destabilization of the interacting heads motif (IHM) by pathogenic HCM variants alters the proportion of myosins in the SRX and DRX conformations in mouse myocardium and human iPSC-CMs.** **A**, Depiction of the functional domains of myosin, subunit 1 (S1), subunit 2 (S2), and light meromyosin (LMM), and the location of human *MYH7* variants in patients with HCM: pathogenic variants (red), variants of unknown significance (VUS) that alter IHM residues (VUS IHM+, orange), and variants that alter residues outside the IHM (VUS IHM–, blue). **B**, The Protein Data Bank 5TBY model showing relaxed paired myosin heads, one with the ATP binding site blocked (olive) or accessible (green). The associated essential light chains (brown and purple), and regulatory light chains (dark blue and blue) are shown. Residues involved in the myosin IHM are depicted as a ribbon, with the location of 3 HCM pathogenic missense variants MYH7^R403Q/+^ (orange), MYH7^V606M/+^ (magenta), and MYH7^R719W/+^ (green). **C**, The same model shown in **B** and depicting the 3 HCM pathogenic missense variants (red), 2 VUS IHM+ (orange), and one VUS IHM– (blue). Two other VUS IHM– studied here are not visible in this projection. **D** and **E**, Proportion of myosin heads in DRX and SRX conformations in WT, Myh6^403/+^, Myh6^606/+^, and Myh6^719/+^ in 8-week-old mouse LV myocardium, in the presence or absence of 0.3 μmol/L MYK-461 as indicated. (See also Figure I in the online-only Data Supplement). **F** and **G**, Proportion of myosin heads in DRX and SRX conformations in WT, MYH7^403/+^, MYH7^606/+^, and MYH7^719/+^ iPSC-CMs in the presence or absence of 0.3 μmol/L MYK-461 as indicated. Data are from 3 independent heart tissues or differentiations of iPSC-CMs and are presented as means±SEM. Significance was tested by 1-way ANOVA and post hoc Bonferroni corrections for significances are denoted in the figure. Significances are reported in relation to WT values; bars encompassing significance values indicate that statistical significance is shared among these samples. DRX indicates disordered relaxed state conformation of myosin molecule; HCM, hypertrophic cardiomyopathy; iPSC-CMs, isogenic cardiomyocytes derived from induced pluripotent stem cells; NS, not significant; SRX, super relaxed state conformation of myosin molecule; and WT, wild type cardiomyocytes.

With evidence that energetic states were associated with proportions of myosins in DRX and SRX conformations, we considered whether cardiac pathologies perturbed these ratios. We previously showed that human HCM pathogenic variants in *MYH7*, which encodes the cardiac β-myosin heavy chain, are clustered in residues that participate in IHM interactions,^12^ an observation that implied that missense residues might destabilize the IHM and thereby alter the balance of DRX and SRX conformations. Residues 403 and 606 reside within the S1 myosin head (Figure 2A) and are positioned along the actin interface, whereas residue 719 resides in the myosin converter domain. Each of these residues is predicted to participate in stabilizing IHM conformations. Residue 719 is also predicted to anchor one myosin head along the partnered myosin’s tail, an interaction that promotes the SRX conformation. HCM variants R403Q and R719Q also cause a –2 change in electrical charge,^12^ which could destabilize these IHM interactions (Figure 2B and 2C). To test these predictions, we initially studied LV myocardium from mice carrying each of these heterozygous variants (R403Q, R719Q, and V606M)^30^ in *Myh6* that encodes the mouse cardiac isoform, α-myosin heavy chain.

We performed Mant-ATP assays on cardiac LV tissue from young *Myh6*^R403Q/+^, *Myh6*^V606M/+^, and *Myh6*^R719W/+^ mice before morphological changes that indicate hypertrophic remodeling. Each mutant heart showed a significantly more rapid Mant-ATP fluorescent decay in comparison with wild type (WT), corresponding to increased proportions of myosins in DRX and decreased in SRX conformations (Figure 2D and 2E and Figure I in the online-only Data Supplement). Application of MYK-461 (see Methods), an allosteric modulator of myosin ATPase activity, normalized the SRX/DRX imbalance in *Myh6*^V606M/+^ hearts, and excessively increased SRX in comparison with DRX conformations in *Myh6*^R403Q/+^ and *Myh6*^R719W/+^ tissues.

Adult human ventricles express primarily β-myosin heavy chain (encoded by *MYH7*), with ≈93% amino acid sequence identity to α-myosin heavy chain, but with 2-fold slower actin sliding velocities and lower ATPase activity.^36^ To assess HCM variants in the context of β-myosin, we engineered heterozygous HCM variants into a *MYH7* allele in human iPSCs that harbor a green fluorescent protein (GFP) tag on titin, so as to allow direct assessment of sarcomere performance.^26,27,31^ Cardiomyocytes derived from iPSC-CMs with heterozygous HCM missense variants were studied at day 30 after differentiation, at which time RNA sequencing demonstrated 20-fold greater expression of *MYH7* than *MYH6* (Table III in the online-only Data Supplement) indicative of cardiomyocyte maturation. Analyses of DRX and SRX conformations in iPSC-CMs with normal *MYH7* sequences had proportions of DRX and SRX conformations similar to those observed in WT mouse LV that express *Myh6.* By contrast, isogenic iPSC-CMs with heterozygous HCM missense variants in *MYH7* (Figure 2F and 2G), like variants in *MYH6*, significantly increased the proportion of myosins in the DRX conformation in comparison with the SRX conformation in comparison with WT cardiomyocytes. Application of MYK-461 to mutant iPSC-CMs consistently normalized SRX/DRX conformations to levels observed in WT iPSC-CMs.

### Myosin Missense Variants in iPSC-CMs Evoke HCM Pathophysiology

**Figure 3.**
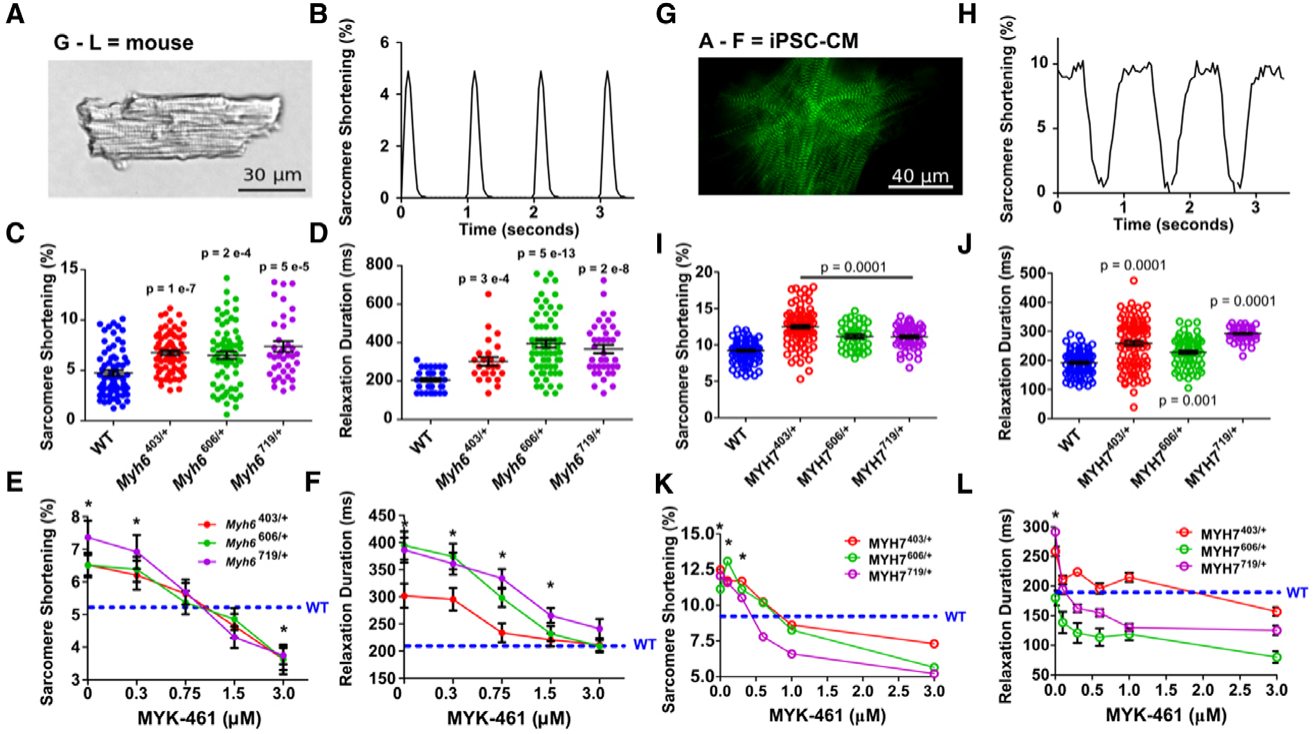
**Pathogenic hypertrophic cardiomyopathy myosin variants in mouse cardiomyocytes and iPSC-CMs exhibit hypercontractility and abnormal relaxation that is normalized by interacting heads motif restabilization with MYK-461.** **A**, Mouse cardiomyocytes (×40 image) shows precise striations attributable to a well-organized sarcomere. **B**, Typical contractile waveform obtained by tracking strings of mouse cardiomyocyte sarcomeres during 1 Hz pacing. **C** and **D**, Sarcomere shortening (**C**) and relaxation durations (**D**) in WT, Myh6^R403Q/+^, Myh6^V606M/+^, and Myh6^R719W/+^ mouse cardiomyocytes taken from 3 hearts per genotype. **E** and **F**, Dose-dependent effects of acute MYK-461 application on sarcomere contractility (**E**, percent shortening) and relaxation (**F**) in paced mouse WT, Myh6^R403Q/+^, Myh6^V606M/+^, and Myh6^R719W/+^ cardiomyocytes from 3 hearts per genotype. **G**, Image of titin-green fluorescent protein–tagged iPSC-CM imaged at 100×, showing *z*-discs labeled by green fluorescence. **H**, Contractile waveform of a single sarcomere defined by a *z*-disc pair during 1 Hz pacing. **I** and **J**, Sarcomere shortening (**I**) and relaxation durations (**J**) in paced isogenic WT, MYH7^R403Q/+^, MYH7^V606M/+^, and MYH7^R719W/+^ iPSC-CMs from 3 separate differentiations. **K** and **L**, Dose-dependent effect of acute MYK-461 application on sarcomere contractility (**K**) and relaxation durations (**L**) in paced isogenic WT, MYH7^R403Q/+^, MYH7^V606M/+^, and MYH7^R719W/+^ iPSC-CMs from 3 separate differentiations. Significant differences to WT were assessed by 2-way ANOVA with post hoc Bonferroni correction from multiple comparisons, significances of *P*<0.05 indicated by *. All data are displayed as mean±SEM. iPSC-CMs indicates isogenic cardiomyocytes derived from induced pluripotent stem cells; and WT, wild type cardiomyocytes.

Isolated mouse cardiomyocytes have highly ordered sarcomeres that propel uniform cellular contraction and relaxation (Figure 3A through 3F) unlike iPSC-CMs. However, iPSC-CMs containing GFP-tagged titin enabled high-fidelity dynamic tracking of hundreds of individual sarcomeres^31^ for direct functional analyses (Figure 3G through 3L). Comparisons of isolated paced cardiomyocytes from WT and HCM mice (Figure 3C) and iPSC-CMs (Figure 3I) showed that each HCM variant caused hypercontractility. In addition, each variant also caused concurrent prolongation in the duration of relaxation in comparison with WT cardiomyocytes (Figure 3D and 3J). Because these changes in functional properties correlated with shifts in the proportions of myosins in DRX and SRX conformations, we deduced that increased contractility likely resulted from increased numbers of myosin in the DRX conformation, whereas delayed relaxation was attributable to decreased numbers of myosin in the SRX conformation. Consistent with this conclusion, application of MYK-461 to mouse cardiomyocytes and iPSC-CMs caused a dose-dependent decrease in the numbers of myosin in DRX and in hypercontractility (Figure 3E and 3K). The prolonged relaxation was normalized to WT rates (Figure 3F and 3L). These data indicate that the overall cardiac effects of MYK-461 may relate to the degree of shifting myosin conformations, as has been suggested from in vitro analyses.^37^

### IHM Destabilization Increases Cell Size and Sarcomere Content and Alters Cellular Metabolism

**Figure 4.**
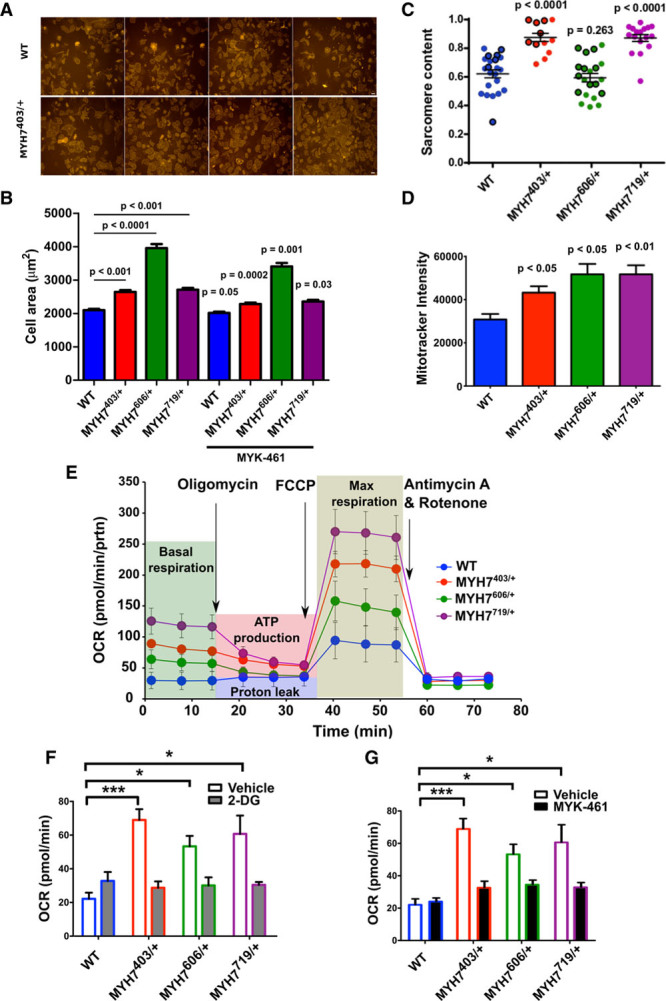
***MYH7* variants affect sarcomere content and cell size in iPSC-CMs.** **A**, Representative images obtained of WT and MYH7^R403Q/+^ iPSC-CMs used for calculating cellular spread area. **B**, Mean cell spreading areas observed with unconstrained seeding onto glass slides of iPSC-CMs: WT (n=632), MYH7^R403Q/+^ (n=530), MYH7^V606M/+^ (n=479), and MYH7^R719W/+^ (n=655), iPSC-CMs (MYH7^R403Q/+^, n=665; MYH7^V606M/+^, n=531; MYH7^R719W/+^, n=653) were treated with 1 μmol/L MYK-461 for 24 hours, and the spreading areas were measured. Data presented are from 3 separate differentiations with the mean±SEM displayed for each measure. Significances are measured by 2-way ANOVA with multiple comparison corrections and a significance cutoff of *P*<0.05. The *P* values above bars compare areas of untreated mutant and WT iPSC-CMs. The *P* values without bars reflect comparisons of MYK-461 treated to untreated cells with the same genotype. **C**, Sarcomere content assessed by Fast Fourier Transform analyses of WT, MYH7^403/+^, MYH7^606/+^, and MYH7^719/+^ iPSC-CMs. Data presented are from 3 separate differentiations with mean±SEM displayed for each measure. Statistical significance was assessed by 1-way ANOVA with post hoc Bonferroni correction for multiple comparisons with a significance cutoff of *P*<0.05. **D**, Mitochondrial abundance assessed by Mitotracker intensity between WT, MYH7^403/+^, MYH7^606/+^, and MYH7^719/+^ iPSC-CMs. **E**, Extracellular-flux analyses during experimental conditions (depicted by background shading) in WT, MYH7^403/+^, MYH7^606/+^, and MYH7^719/+^ iPSC-CMs. Assessment of oxygen consumption rate (OCR) was performed with the application of compounds to assess ATP production (Oligomycin), maximum (Max) respiration (FCCP, carbonyl cyanide-4 [trifluoromethoxy] phenylhydrazone) and halted respiration (antimycin A and rotenone) for WT or mutant MYH7^403/+^, MYH7^606/+^, MYH7^719/+^ iPSC-CMs. **F**, Basal OCR of naive WT MYH7^403/+^, MYH7^606/+^, and MYH7^719/+^ iPSC-CMs or after 24-hour MYK-461 0.3 μmol/L treatment. **G**, Basal OCR of naive WT MYH7^403/+^, MYH7^606/+^, and MYH7^719/+^ iPSC-CMs or after 2-deoxy-d-glucose (2-DG) treatment. * denotes significance of *P*<0.05, *** denotes a significance of *P*<0.005. iPSC-CMs indicates isogenic cardiomyocytes derived from induced pluripotent stem cells; and WT, wild type cardiomyocytes.

On the basis of the demonstration that pathogenic *MYH7* missense variants, which destabilized the IHM, caused functional phenotypes (hypercontractility and poor relaxation), we considered whether or not IHM destabilization also affected cellular morphology and energetics. Hypertrophy, a prominent clinical component of HCM, was assessed by comparing cell sizes of isogenic WT and mutant iPSC-CMs (Figure 4A and 4B). The mean area of unconstrained mutant cells was significantly larger than WT cells (MYH7^R403Q/+^, *P*<0.001; MYH7 ^V606M/+^, *P*<0.0001; MYH7^R719W/+^, *P*<0.001). In comparison with untreated mutant iPSC-CMs, exposure of cells to 1 μmol/L MYK-461 for 24 hours caused significantly reduced cell areas (Figure 4B). We assessed sarcomere content by fluorescence-activated cell sorting analysis of GFP-tagged titin iPSCs before differentiation (Figure II in the online-only Data Supplement) and in mature iPSC-CMs by Fast Fourier Transform analyses (Figure 4C). In comparison with WT iPSC-CMs, sarcomere content was increased in MYH7^R403Q/+^and MYH7^R719W/+^ iPSC-CMs and not in MYH7^V606M/+^ (Figure 4C).

Given increased size and sarcomere content and the increased proportions of mutant myosins in DRX that would propel increased contractility and 5-fold greater ATP consumption,^11^ we assessed cellular energetics. Mitochondrial content was increased in mutant lines (Figure 4D). Assays of cellular OCRs showed that basal rates of OCR were increased in mutant compared with WT iPSC-CMs, and mutant iPSC-CMs had greater capacity for maximal cellular respiration than that achieved by WT cells (Figure 4E). Because the addition of the glucose analogue, 2-deoxy-d-glucose, markedly decreased oxygen consumption rates in mutant in comparison with WT iPSC-CMs (Figure 4F), we deduced that mutant cells used glycolysis to supplement energy requirements. Acute treatment of iPSC-CMs with MYK-461 (Figure 4G) restored normal OCR (versus WT, *P*=not significant). We concluded from these data that increased cellular energy demands were driven by IHM destabilization, which increased myosin proportions in DRX. Pharmacological rebalancing of DRX:SRX proportions removed the increased energetic burden in cardiomyocytes.

**Figure 5.**
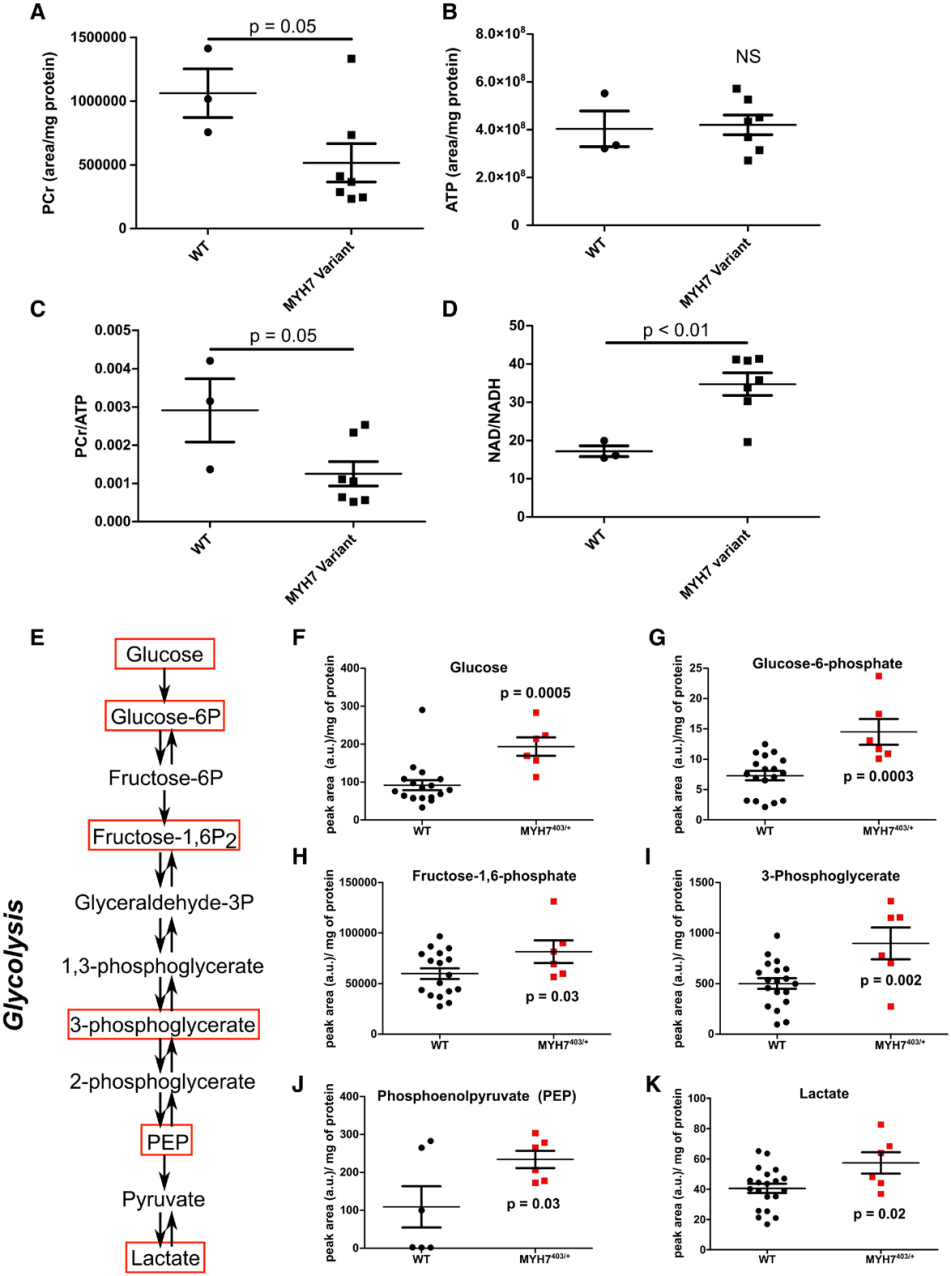
***MYH7* variants that destabilize the interacting heads motif alter cellular metabolism.** **A**, Mass spectrometry assessment of total cell phosphocreatine (PCr) abundance in WT, and combined MYH7^403/+^, MYH7^606/+^, and MYH7^719/+^ iPSC-CMs. **B**,Mass spectrometry assessment of total cell adenosine triphosphate (ATP) abundance in WT, and combined MYH7^403/+^, MYH7^606/+^, and MYH7^719/+^ iPSC-CMs. **C**, Mass spectrometry assessment of total cell PCr/ATP in WT, and combined MYH7^403/+^, MYH7^606/+^, and MYH7^719/+^ iPSC-CMs. **D**, Mass spectrometry assessment of total cell NAD/NADH in WT, and combined MYH7^403/+^, MYH7^606/+^, and MYH7^719/+^ iPSC-CMs. **E**, Analyses of metabolic pathways involved in glycolysis. Glycolytic intermediates marked with red boxes were assessed by mass spectrometry. **F** through **K**, Glycolytic pathway intermediates detected in WT and MYH7^403/+^ iPSC-CMs. (See also Figure III in the online-only Data Supplement). All data were obtained from day 30 cultures of ≥2 independent differentiations, per genotype, and are displayed as mean±SEM. One-way ANOVA and 2-way ANOVA with post hoc Bonferroni correction for multiple comparisons were used for **A** and **C** and **D**, respectively. *t* tests defined statistical significance in **F** through **K**. a.u. indicates arbitrary units; Fructose-1,6-P_2_, fructose 1,6-bisphosphate; iPSC-CMs, isogenic cardiomyocytes derived from induced pluripotent stem cells; NS, not significant; PEP, phosphenolpyruvate; and WT, wild type cardiomyocytes.

We considered whether ATP levels were altered in MYH7 variants or were stable because of supplemental sources that accommodated increased energy requirements.^38^ Using mass spectrometry, we compared WT and mutant iPSC-CMs to assess phosphocreatine and ATP levels and phosphocreatine/ATP and NAD/NADH ratios. In comparison with WT cells, mutant iPSCs had reduced phosphocreatine levels (*P*=0.05, Figure 5A) but stable ATP levels (Figure 5B), likely because of significantly reduced phosphocreatine/ATP levels (*P*=0.05, Figure 5C). In addition, mutant iPSC-CMs had increased NAD/NADH ratios (*P*<0.01, Figure 5D). Because our analyses showed that 2-deoxy-d-glucose–treated iPSC-CMs reduced OCR, we considered if increased glycolysis (Figure 5E) contributed to these changes. Cardiomyocytes preferentially metabolize fatty acid oxidation for ATP production, but use glycolysis to supplement energetic demands, in particular, in response to stresses.^39,40^ Using tandem mass spectrometry to assess metabolomics, we found that, in comparison with WT, MYH7^R403Q/+^ iPSC-CMs had increased levels of metabolomic by-products associated with active glycolysis (Figure 5F through 5K), as did LV tissues from Myh6^R403Q/+^ and Myh6^R719W/+^ in comparison with WT mice (Figure III in the online-only Data Supplement). Consistent with the increased mitochondria content and OCR levels, tricarboxylic acid metabolites were significantly increased in MYH7^R403Q/+^ iPSC-CMs (Figure III in the online-only Data Supplement).

Together these experiments link destabilization of the IHM by pathogenic myosin variants with altered proportions of DRX and SRX conformations, hypercontractility, poor relaxation, hypertrophic remodeling of cardiomyocytes, increased mitochondrial content, and energetic and metabolic stress. Because pharmacological normalization of SRX/DRX ratios ameliorated downstream functional abnormalities, we conclude that the physiological regulation of myosins in DRX and SRX conformations is central to cardiac homeostasis, and that chronic dysregulation of myosin conformations is the fundamental pathophysiologic basis for HCM.

### IHM Destabilization Predicts Adverse Clinical Outcomes

Because IHM destabilization defined a cellular mechanism by which 3 pathogenic *MYH7* variants cause HCM, and because restabilization of the IHM subverted these cellular phenotypes, we considered whether additional *MYH7* amino acid substitution, specifically variants with unknown significance (VUS) also destabilized the IHM. Using these data, we assessed associations with clinical outcomes of the patients with HCM.

**Figure 6.**
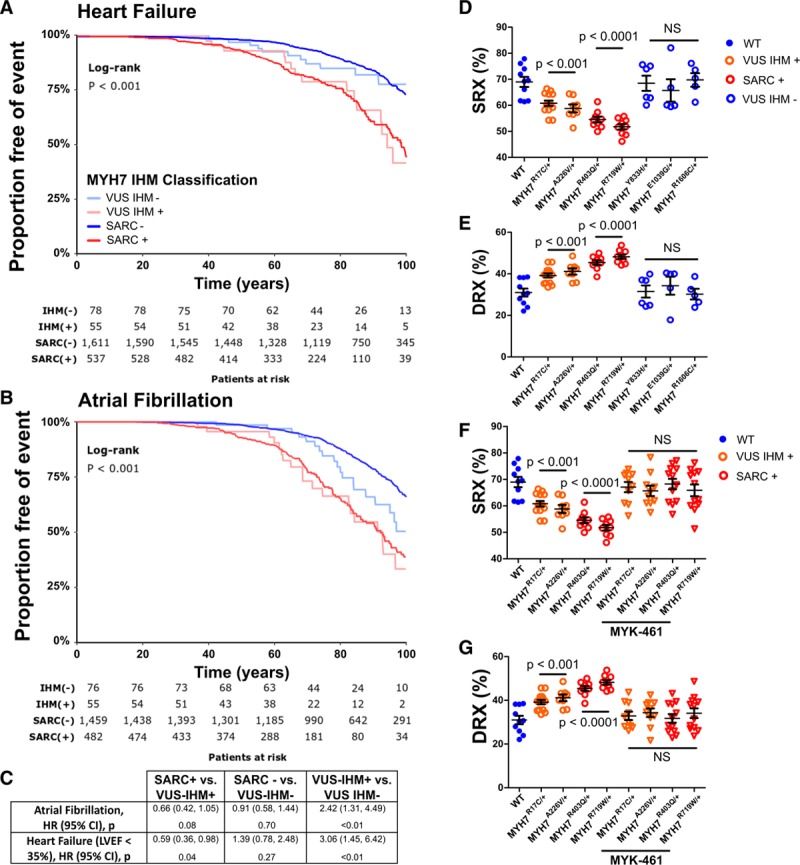
**Myosin VUS that alter IHM residues are associated with higher rates of adverse clinical outcomes.** **A**, Kaplan-Meier curves show relative time points at which SHaRe subjects, grouped according to genotype, meet composite heart failure end points (first occurrence of cardiac transplantation, LV assist device implantation, LV ejection fraction <35%, or New York Heart Association class III/IV symptoms). Genotypes are denoted as pathogenic sarcomere variants (SARC+), no pathogenic sarcomere variants (SARC–), variant of unknown significance that alters an IHM residue (VUS–IHM+), and variant of unknown significance that does not alter an IHM residue (VUS–IHM–). **B**, Kaplan-Meier curves demonstrate time to atrial fibrillation among SHaRe subjects with genotypes SARC+, SARC–, VUS–IHM+, and VUS–IHM–. (See also Figure IV in the online-only Data Supplement). **C**, Cox proportional hazard ratios comparing clinical SHaRe subjects with genotypes SARC+, SARC–, VUS–IHM+, and VUS–IHM– and adverse clinical outcomes of heart failure and atrial fibrillation. Significance was calculated using the log-rank test. **D** and **E**, Proportion of myosins in the SRX (**D**) and DRX conformations (**E**) in human ventricular tissues from healthy donor tissues (WT) or from HCM subjects with genotypes SARC+, VUS IHM+, (SARC–), and VUS IHM–. **F** and **G**, Proportion of myosin heads in SRX (**F**) and DRX (**G**) from healthy human ventricular tissues (WT) or HCM hearts with genotypes SARC+, VUS IHM+, (SARC–), and VUS IHM– region (VUS IHM +) before or after treatment with 0.3 μmol/L MYK461. All data are displayed with mean±SEM, and all significances are reported with a significance cutoff of *P*<0.05 after using two-way ANOVA with Bonferroni corrections for multiple comparisons. DRX indicates disordered relaxed state conformation of myosin molecule; HCM, hypertrophic cardiomyopathy; HR, hazard ratio; IHM, interacting-heads motif; LV, left ventricular; LVEF, left ventricular ejection fraction; NS, not significant; SHaRe, Sarcomeric Human Cardiomyopathy Registry; and SRX, super relaxed state conformation of myosin molecule.

A clinical study of >4500 patients with HCM enrolled in the SHaRe showed that participants with pathogenic variants in sarcomere protein genes had the highest rates of heart failure and atrial fibrillation.^14^ However, many human *MYH7* missense variants that are identified in patients with HCM cannot be definitively classified as pathogenic or benign and are designated VUS. Among SHaRe participants, the rates of heart failure and atrial fibrillation among patients with sarcomere VUS was intermediate, below that observed in patients with pathogenic sarcomere variants (SARC+), but higher than in patients without sarcomere variants (SARC–). We hypothesized that the intermediate risk associated with VUS resulted from poor annotation of variants and the combined outcomes of patients with variants that were actually pathogenic and patients with benign variants. To test this model, we reclassified *MYH7* VUS, on the basis of whether the variant altered residues participating in the IHM (VUS–IHM+) or not (VUS–IHM–). We then asked if the lifetime burden of heart failure and atrial fibrillation differed between patients who had HCM with VUS–IHM+ and VUS–IHM– by comparing these outcomes in 2281 patients with HCM stratified by genotype: 537 with pathogenic variants, 55 with VUS–IHM+, 78 with VUS–IHM–, and 1611 without sarcomere variants (Figure 6A and 6B and Figure IV in the online-only Data Supplement). Rates of heart failure and atrial fibrillation were significantly higher in patients in the VUS–IHM+ group than those in the VUS–IHM– and SARC– groups (Figure 6A through 6C).

We corroborated these observational studies by Mant-ATP assays of human cardiac ventricular tissues from patients with HCM (See Table II in the online-only Data Supplement for details) with pathogenic variants (MYH7^R403Q/+^ and MYH7^R719W/+^), VUS–IHM+ (MYH7^R17C/+^ and MYH7^A226V/+^), and VUS–IHM– (MYH7^Y833H/+^, MYH7^E1039G/+^, and MYH7^R1606C/+^). Both VUS–IHM+ and one VUS–IHM– (MYH7^Y833H/+^) reside within the S1 domain of myosin, as do both pathogenic variants (Figure 2A and 2C). Two other VUS–IHM– reside in the myosin light meromyosin domain (Figure 2A). Similar to pathogenic HCM variants, cardiac tissues with either VUS–IHM+ increased the proportion of myosins in DRX in comparison with SRX conformations (Figure 6D through 6G). By contrast, the proportion of myosins in DRX and SRX conformations in VUS–IHM– tissues were comparable to the physiological levels found in the healthy human heart. Application of MYK-461 to human heart tissues from patients with VUS–IHM+ or with pathogenic variants MYH7^R403Q/+^ and MYH7^R719W/+^ normalized the SRX/DRX ratios (Figure 6F and 6G).

## Discussion

The necessity for continual contractile performance by the heart mandates extraordinary sarcomere efficiency, matching power output with energy supplies, especially because cardiomyocytes have virtually no self-renewal and must continue to beat 2 billion times in the average lifetime. To accomplish this feat, the human heart is estimated to consume 6 kg of ATP each day,^41^ a substantive energy requirement that is predominantly attributed to sarcomere contraction.^42^ By integrating structural, biophysical, biochemical, and clinical information, we provide evidence that dynamic conformations of myosin molecules are central to balancing muscle work and metabolic costs that impact sarcomere function, efficiency, and patient outcomes. We found that hibernating mammals, with limited energy stores, increase the proportion of myosins in the SRX conformaton in comparison with active myosins as a physiological adaptation to reduce the energy demands, and contractility of myocardium during torpor. Reciprocally, human HCM mutations that reduced the proportion of myosin in the SRX conformation resulted in increased contractility at the expense of higher energetic demands and impaired relaxation. Restoration of appropriate proportion of myosin in the SRX conformation by an allosteric myosin modulator normalized biophysical and metabolic abnormalities, providing evidence for the primacy of myosin conformations in sarcomere efficiency and cellular homeostasis.

Our analyses of *MYH7* VUS confirmed the importance of the IHM in enabling dynamic conformational changes in myosins. Only VUS that destabilized the IHM interactions had increased proportions of myosins in DRX, and patients with these VUS had high rates of heart failure and atrial fibrillation, comparable to those observed in patients with pathogenic HCM mutations. These data support the relevance of leveraging IHM structural models to improve molecular prediction of clinically actionable human variants.

The myosin phylogeny is highly evolutionarily conserved, in particular, myosin II molecules that include ancient myosins, and cardiac myosins, as well.^25^ These ATP-fueled motor proteins are found in muscle and nonmuscle cells and function in cell division, migration, contraction and relaxation, endocytosis and exocytosis, and cell shape. Evolutionary conservation of protein motifs has implied functional importance of myosin domains involved in binding actin, ATP hydrolysis, and interactions with other proteins. We suggest that this evolutionary conservation extends to IHM residues, based on analyses of mouse LV (predominantly Myh6 protein) and human LV (predominantly MYH7 protein). The IHM motif enables myosin head-head and head-tail interactions that promote or limit actomyosin engagement and ATP hydrolysis. That these conformations that are conserved across species evolution strongly infers that efficient regulation of myosin activity and ATP consumption is critical.

We demonstrate the cellular effects of physiological dysregulation of myosin conformations. Hibernating ground squirrels had 10% more cardiac myosins in the SRX conformation than when squirrels were active. Because myosins in the SRX conformation consume 5-fold less ATP,^11^ this shift in myosin conformations is predicted to provide a 17.5% reduction in energy usage, a considerable savings for fuel-starved hibernating animals. We observed a similar degree of conformational shift in myosin with HCM variants, albeit with increased proportion of myosins in the DRX, not SRX conformation. Because pathogenic *MYH7* variants are expressed lifelong, this shift would chronically increase energy usage and a substantial metabolic burden on cardiomyocytes. Moreover, because our experiments in unloaded hearts and cells that could underestimate these effects, we expect that physiological and pathological hemodynamic stress would also cause conformational shifts in myosins that further exacerbate energy depletion. Consistent with these observations, mutant iPSC-CMs had increased mitochondrial content, and, despite this, adopted a stress-induced transcriptional and metabolic profile, using glycolysis, and fatty acid oxidation, as well, to generate ATP.

Although our experiments were focused at the level of cardiomyocytes, these data may explain multiple in vivo phenotypes of HCM: hyperdynamic contraction, poor relaxation, hypertrophy, and increased energy consumption. Although hypertrophic remodeling increases LV wall thickness and reduces cavity volumes, hyperdynamic contraction and poor relaxation predates these morphological changes.^43^ Consistent with these observations, the coincident increase in myosin DRX proportions and cardiomyocyte systolic shortening observed here indicates that hypercontractility is a direct biophysical response to HCM mutations. Although we did not assess calcium sensitivity iPSC-CMs, which have immature development of the calcium-handling apparatus, hyperdynamic contraction may reflect primary or secondary increases in calcium sensitivity, as has been observed in HCM mouse models^30^ and human HCM tissues.^44^ Chronic hyperdynamic contraction might also contribute to hypertrophy, similar to the effects of conditioning exercises on muscle mass of highly trained athletes.^45^ Concurrent with the increased proportion of myosins in DRX, the reduced proportions of myosins in SRX conformations are likely to be an important cause for poor relaxation in HCM hearts that contributes to patients’ outcomes. Impaired ventricular relaxation and compliance impedes atrial emptying that, when chronic, leads to dilation of the thin-walled atrial chamber to promote atrial fibrillation, an arrhythmia that occurs in >20% of patients with HCM.^46^

Energetic abnormalities are also prominent in patients with HCM^47^ and precede morphological phenotypes of cardiac hypertrophy,^48^ indicating that these too are proximal consequences of mutations. The association of shifting mutant myosins toward DRX with profound deficits in normal metabolic profiles in mutant iPSC-CMs^22^ provided a plausible explanation for these early energetic abnormalities, although more comprehensive analyses are warranted. The heart depends on efficient ATP production from mitochondrial oxidative phosphorylation, which is estimated to supply 95% of the myocardial ATP requirements.^49^ Although the heart can activate glycolysis to acutely meet increased energetic demands, a shift away from use of fatty acid substrates occurs at the cost of diminished efficiency in ATP production, and this metabolic reprograming is a recognized mechanism for heart failure.^50^ As such, the increased rates of heart failure in patients who have HCM with either pathogenic mutations or VUS that disrupt the IHM support our conclusion that myosin conformations are central regulators of cardiomyocyte metabolism. In addition, these data provide the opportunity to recognize patients at highest risk for heart failure, a substantial contributor to premature death in HCM.^14^

In summary, demonstration that pathogenic *MYH7* variants destabilize the IHM not only alter the mechanical properties of the sarcomere, but also incite profound changes in cardiomyocyte physiology, provides mechanistic insights into the major clinical manifestations of HCM: hypertrophic remodeling, hypercontractility, poor relaxation, and excessive energy consumption. Because these cellular phenotypes are the likely underpinning of atrial fibrillation and heart failure, strategies to restore physiological regulation of myosin conformations may limit these adverse clinical outcomes in patients with HCM.

## Sources of Funding

Support for this study was provided in part by the Wellcome Trust (to Dr Toepfer: Sir Henry Wellcome Fellowship 206466/Z/17/Z), Sarnoff Foundation (to Drs Garfinkel and Repetti), Engineering Research Centers Program of the National Science Foundation (to J.F. Ewoldt, P. Cloonan, and Drs Chopra, Chen, J.G. Seidman, and C.E. Seidman: National Science Foundation Cooperative Agreement No. EEC-1647837), an unrestricted research grant from MyoKardia Inc (to Drs Olivotto, Colan, Ashley, Jacoby, Michels, Day, and Ho), the Italian Ministry of Health (RF-2013-02356787), NET-2011-02347173; and FAS-Salute 2014, Regione Toscana (to Dr Olivotto), American Heart Association and Taubman Medical Institute (to Dr Day), British Heart Foundation (RG/12/16/29939) and British Heart Foundation Centre of Research Excellence (Oxford; to Dr Watkins and Redwood), Fondation Leducq (to Drs J.G. Seidman and C.E. Seidman), the National Institutes of Health (to Dr Ho: 1P50HL112349 and 1U01HL117006; to Dr Day: HL11572784; to Dr J.G. Seidman: U01HL098166; to Drs J.G. Seidman and C.E. Seidman: 5R01HL080494 and 5R01HL084553), Sãto o Paulo Research Foundation (Drs Pereira and Venturini.: 2017/20593-7), and the Howard Hughes Medical Institute (to Drs C.E. Seidman and Padrón).

## Disclosures

Drs C.E. Seidman and J.G. Seidman are founders and own shares in MyoKardia Inc, a startup company that is developing therapeutics that target the sarcomere. MyoKardia had no role in performing or approving these studies. The other authors report no conflicts.

## Supplementary Material


