# Supplementary material for: Myosin Sequestration Regulates Sarcomere Function, Cardiomyocyte Energetics, and Metabolism, Informing the Pathogenesis of Hypertrophic Cardiomyopathy
Source: Circulation. 2020 Jan 27;141(10):828–42. doi: 10.1161/CIRCULATIONAHA.119.042339 (PMC7077965; doi:10.1161/CIRCULATIONAHA.119.042339)
Supplement: Supplementary file 1 [file cir-141-828-s001.pdf]

Online Data Supplement

Myosin sequestration regulates sarcomere function, cardiomyocyte energetics and metabolism, informing the pathogenesis of hypertrophic cardiomyopathy

Christopher N. Toepfer, Amanda C. Garfinkel, Gabriela Venturini, Hiroko Wakimoto, Giuliana Repetti, Lorenzo Alamo, Kallyandra Padilha, Victor C. Bellini, Arun Sharma, Radhika Agarwal, Jourdan F. Ewoldt, Paige Cloonan, Justin Letendre, Mingyue Lun, Iacopo Olivotto, Steve Colan, Euan Ashley, Daniel Jacoby, Michelle Michels, Charles S. Redwood, Hugh C. Watkins, Sharlene M. Day, James F. Staples, Raúl Padrón, Chopra A, Carolyn Y. Ho, Christopher S. Chen, Alexandre C. Pereira, Jonathan G. Seidman[^], and Christine E. Seidman[^*]

Correspondence to: Christopher_toepfer@hms.harvard.edu

cseidman@genetics.med.harvard.edu

This PDF file includes **Supplemental Methods**

Supplemental Table 1-2

Supplemental Figures 1 -4

Supplemental References

Expanded Methods

Rodent studies

Mouse cardiomyocytes were isolated from 8-week old mouse hearts using Langendorff apparatus for perfusion with Enzyme Buffer (EB composition: 135 mM NaCl, 4 mM KCl, 0.33 mM NaH_2PO_4, 1 mM $MgCl_2$, and 10 mM HEPES, pH 7.40, which incorporated Collagenase D, Collagenase B and Protease XIV) for 10 minutes. After perfusion the atria and right ventricle were removed and the left ventricle was minced in TA buffer (Composition: 135 mM NaCl, 4 mM KCl, 0.33 mM NaH_2PO_4, 1 mM $MgCl_2$, and 10 mM HEPES, pH 7.40, which incorporated bovine serum albumin) and passed through a 100 µm filter into a 50ml conical tube. Tissue settled for 15 minutes to allow cardiomyocytes to pellet by gravity. The pellet was then sequentially resuspended every 10 minutes through an increasing calcium gradient (5%, 20%, 50%, 100% calcium tyrode) to provide a cell fraction enriched in myocytes with a final experimental buffer (Composition: NaCl 137 mM, KCl 5.4 mM, $CaCl_2$ 1.2 mM, $MgCl_2$ 0.5 mM, HEPES 10 mM at pH 7.4, which incorporated glucose). Functional analyses were performed as described previously [1].

Contractile measurements of mouse cardiomyocytes

Cardiomyocytes were placed in a 6-well plate, precoated with laminin for two hours at a concentration of 10 ug/mL in PBS (Composition: KH_2PO_4 1mM, NaCl 155 mM, Na_2HPO_4 3mM, at pH 7.4). Cells were imaged using a Keyence BZ-X710 microscope using a Nikon 40X/0.65 NA objective. Cells were kept at 37°C using a humidified incubation chamber that delivered 20% O_2 and 5% CO_2. Cells were paced at 1Hz using custom-built electrodes with a pacing unit (Pulsar 6i, FHC Brunswick, ME, USA) delivering 20V. Movies were acquired at 29 frames per second for 5 seconds.

SarCoptiM was used to track sarcomere lengths during contractile cycles [2]. Sarcomere tracking provided measures of cellular shortening (%), relaxed and contracted sarcomere lengths (µm), contractile cycle and relaxation durations (seconds).

For experiments incorporating MYK-461, drug was applied in concentrations ranging 0.3 – 3 µM in the experimental buffer. MYK-461 was incubated with cells for a minimum of 10 minutes before data acquisition.

Mant-ATP assays of myocardial tissues

Approximately 20 mg mouse or human left ventricular myocardium was permeabilized on ice for 6 hours in permeabilization buffer (Composition: NaCl 100 mM, $MgCl_2$ 8 mM, EGTA 5 mM, K_2HPO_4 5 mM, KH_2PO_4 5 mM, NaN_3 3 mM, ATP 5 mM, DTT 1 mM, BDM 20 mM, Triton-X 100 0.1%, at pH 7.0). Samples were subsequently stored overnight at -20°C in glycerinating solution (Composition: K acetate, 120 mM; Mg acetate, 5 mM; K_2HPO_4, 2.5 mM; KH_2PO_4, 2.5 mM; MOPS, 50 mM; ATP, 5 mM; BDM, 20 mM; DTT, 2 mM; glycerol, 50% (v/v), pH 6.8.) for use within 2 days.

Myocardium was dissected into ~ 90 x 400 µm pieces, held under two pins in a flow chamber constructed from a slide and coverslip. Samples underwent permeabilization using the permeabilization buffer for 30 minutes on ice. After secondary permeabilization chambers were flushed with glycerinating buffer.

Chambers were imaged using a Nikon TE2000-E was used with Nikon 20X/0.45 objective, using a Hammamatsu ORCA-flash4.0 EM-CCD. For 15 minutes, stacked images were acquired with exposures every 10 seconds, with a 10 ms acquisition and exposure time using a DAPI filter set. Experiments were performed by initially flushing ATP buffer (Composition: K acetate 120 mM, Mg acetate 5 mM, K_2HPO_4 2.5 mM, KH_2PO_4 2.5 mM, ATP 4 mM, MOPS 50 mM, DTT 2 mM at pH 6.8) to remove glycerol, which was replaced with rigor buffer (Composition: K acetate 120 mM, Mg acetate 5 mM, K_2HPO_4 2.5 mM, KH_2PO_4 2.5 mM, MOPS 50 mM, DTT 2 mM at pH 6.8). Rigor buffer was incubated for 5 minutes, and initial fluorescence acquisition was simultaneous with the addition of rigor buffer with 250 µM Mant-ATP to visualize fluorescent Mant-ATP wash in. At the end of the 15-minute image acquisition, a chamber volume of ATP buffer (Rigor buffer + 4 mM ATP) was added to the chamber with simultaneous acquisition of the Mant-ATP chase. For experiments with MYK-461 all experimental solutions contained 0.3 µM MYK-461.

Human Studies

Human subjects participating in Sarcomeric Human Cardiomyopathy Registry (SHaRe) were recruited after obtaining written signed consent using protocols that were reviewed and approved by each institution of the participating SHaRe investigators, as previously described[3]. The races and ethnicities of the SHaRe participants studied here are provided in the **Supplemental Table 1**. SHaRe participants were required to have a site-designated diagnosis of HCM, defined as unexplained LV hypertrophy with maximal LV wall thickness > 13mm (or equivalent

Z-score for pediatric patients), without accompanying extracardiac features, family history. Additional inclusion criteria required ≥1 clinic visit at a SHaRe site since 1960, ≥1 echocardiographic assessment of LV wall thickness, and availability of genetic test data including evaluation of the 8 sarcomere gene mutations associated with HCM (myosin binding protein C (*MYPBPC3*), myosin heavy chain (*MYH7*), cardiac troponin T (*TNNT2*), cardiac troponin I (*TNNI3*), α-tropomyosin (*TPM1*), myosin essential and regulatory light chains (*MYL3, MYL2*), and actin (*ACTC*), as previously described[3].

Clinical outcomes in SHaRe participants were studied using Kaplan-Meier analyses to compare time to event (atrial fibrillation and heart failure) by genotype groups. Because sarcomere mutations are present from birth and there is rigorous ascertainment of events that occurred prior to initial evaluation at a SHaRe site, events were analyzed from birth to assess the lifetime cumulative incidence of adverse clinical outcomes. Patients who did not have the outcome of interest were censored at the time point of their last recorded visit in SHaRe, and patients who lacked data on occurrence or timing of the outcome of interest were excluded from analysis of that particular outcome.

SHaRe participants with genotypes, including those with a pathogenic or likely pathogenic variant (SARC +) those without such variants (SARC-), and those with a variant of unknown significance (VUS) that altered residues participating in the IHM (VUS-IHM+) or that altered residues outside of the IHM ((VUS-IHM-) were studied. The ages at first event were compared for patients stratified by these genotypes, using hazard ratios based on Cox proportional hazards models with family-specific frailty effects to account for correlation due to relatedness. The proportional hazards assumption was checked using statistical tests and graphical diagnostics based on the scaled Schoenfeld residuals. As a plot of the Schoenfeld residuals against time for each covariate did not show evidence of a non-random pattern, there was no evidence for a violation of the proportional hazards assumption.

Mant-ATP analyses of Human LV Tissues

Discarded human left ventricular heart samples (**Supplemental Table 2**) were obtained from a subset of SHaRe participants who underwent procedures for clinical indications at Brigham and Women's Hospital or the University of Michigan Medical Center. The myocardial location of the sample, patient's demographics, genotype,

assessment of cardiac function, and indication for surgery were obtained. Tissues were snap-frozen in liquid N_2 immediately upon excision.

Three regions of each experiment were sampled for fluorescence decay using the ROI manager in ImageJ as described previously [1, 4]. The final data point of fluorescence wash in defined the y-intercept. Subtraction of non-myosin bound Mant-ATP fluorescence was corrected as indicated previously[5]. All data presented as normalized intensity of peak fluorescent intensity. These data are fit to an unconstrained double exponential decay using Sigmaplot:

$$\text{Normalized Fluorescence} = 1 - A1\,(1-\exp^{(-t/T1)}) - A2\,(1-\exp^{(-t/T2)})$$

Where A1 is the amplitude of the initial rapid decay approximating the disordered relaxed state (DRX) with T1 as the time constant for this decay. A2 is the slower second decay approximating the proportion of myosin heads in the super relaxed state (SRX) with its associated time constant T2.

Each individual experiment was fit using this double exponential decay with all values determined and plot. Statistical analysis was performed using 2-way ANOVA with multiple comparisons tests.

Myosin Isoform Expression iPSC-CMs

The levels of myosin isoform transcripts were assessed in multiple cultures of day 30 iPSC-CMs derived from ≥ three independent differentiations **(Online Data, Supplemental Table 3**). Cells were harvested in Trizol (Thermo Fisher) and extracted RNA was used for library preparation via the Nextera method. Libraries were sequenced on a HiSeq 2000 (Illumina) and raw reads were aligned by HISAT2 to human genome (hg38). Aligned reads were quantitated by FeatureCounts, counts were normalized, and the *MYH6* and *MYH7* transcripts were quantified, as described previously[6-8].

Myosin protein isoforms were also assessed in day 30 iPSC-CMs using mass spectrometry (MS). Whole-cell lysates were prepared using previously described methods[9]. In brief , 100μg of total protein for each sample was digested with trypsin and Lyc-C proteases, subjected to Tandem Mass Tag Labeling. Samples were pooled at 1:1:1 ratio and fractionated using basic reversed phase HPLC and subjected to MS/MS analysis using an Orbitrap Fusion Lumos mass spectrometer (Thermo Fisher Scientific, San Jose, CA) coupled to a Proxeon EASY-nLC 1200 liquid chromatography (LC) pump (Thermo Fisher Scientific). Mass spectra were processed using MaxQuant as described

previously[10]. Spectra were converted to mzXML using a modified version of the ReAdW.exe. database. Searching included all entries from the human UniProt database. iBAQ values (sum of peak intensities of all peptides matching to a specific protein divided by the number of observable peptides of a protein to derive the total protein levels) were estimated using MaxQuant, as previously described[11]).

Contractile measures of iPSC-CMs using SarcTrack

For SarcTrack analyses, GFP-tagged iPSC-CMs (usually day 14 post differentiation) were replated onto 12-well plates (or 96-well plate for drug treatments, MatTek) containing 1:100 Matrigel (Corning) in RPMI 1640 medium. Two days after replating, cells were returned to RPMI containing B27, with insulin, subsequently changed every two days. Imaging was performed at day 30 post-differentiation of iPSC-CMs, a commonly used time-point for functional analyses.

MYK-461 from Cayman Chemical was dissolved in DMSO (10 mM) and stored at -80°C. MYK-461 was applied (day 30 post-differentiation) in 96-well imaging plates diluted in RPMI 1640 plus B27 and insulin medium in doses ranging from 0-3 μM final concentrations. 96-well imaging plates containing iPSC-CMs, were equilibrated at 37°C and 5% CO_2 for one hour prior to imaging.

Small iPSC-CM clusters were imaged at 29 frames per second for 5 seconds, under 1Hz pacing. For contractility analysis SarcTrack[12] was used running image stacks on the Harvard Medical School high-performance computing cluster.

Extracellular flux analyses

Cells were washed with prewarmed XF media (non-buffered DMEM supplemented with 2 mM sodium pyruvate, 4mM L-glutamine and 10 mM glucose, pH 7.4, and incubated at 37 °C for 60 min without CO_2. Basal levels of OCR and ECAR were measured over 18 minutes, followed by a mitochondrial stress test adding 2 μM of oligomycin, 2 μM FCCP, 0.5 μM rotenone/0.5 μM antimycin A. After the assay, cells were lysed with RIPA buffer and protein content was measured by BCA Assay (Pierce). Data was analyzed using the WAVE software (Agilent).

Metabolite extraction and derivatization

WT and MYH7$^{403/+}$ iPSC-CM (1.5x10^6 cells) at day 30 post-differentiation were washed with cold PBS and scraped with 1mL of Acetonitrile:Isopropanol:Ultra-Pure water solution (3:3:2 vol/vol). The mixture was centrifuged 15800xg at 0°C for 5 minutes. Five microliters of internal standard myristic acid D27 (3mg/mL) and succinic acid D4 (10µg/mL) were spiked in each sample. Supernatant was dried on speedvac for 18 hours. Metabolites was derivatized using 20µL of methoxyamine/pyridine solution (40mg/mL), followed 90µL of MSTFA with 1% TMCS [13].

Gas chromatography and untargeted mass spectrometry analyses

One microliter of each sample was injected into an Agilent 7890B GC system in splitless mode. A DB5-MS + 10m Duraguard capillary column was used at a helium gas rate of 0.89mL min -1. The column temperature was held at 60ºC for 1 min, and then increased to 310ºC at a rate of 10ºC/min for 37 minutes. The detector (Agilent 5977A) operated in the electron impact ionization mode (70 eV) and mass spectra were recorded after a solvent delay of 6.5 min. Mass spectrometry operated in scan mode with 2.9 scans per second, starting at mass 50 and ending at mass 550, with step size of 0.1m/z. Data consistency was checked based on the identification of Internal Standard (Myristic Acid D27) among samples, identification of each metabolite in at least 2 of 3 technical replicates and a coefficient variation of metabolites intensity lower than 30% among the 3 technical replicates. Only metabolites that were present in at least 50% of samples from one group (WT and MYH7$^{403/+}$) were submitted to statistical analysis. Metabolites were analyzed using the MetaboAnalyst online platform [14]. For missing values, we inserted half of the lower value in the table, and data were normalized using auto-scaling method. Metabolite intensities were log2 transformed and t test was performed with FDR correction for multiple testing.

Gas chromatography and targeted mass spectrometry analysis

Targeted metabolomics was performed according to Calderon-Santiago [15]. One microliter of each sample was injected into an Agilent 7890B GC system in splitless mode. A DB5-MS + 10m Duraguard capillary column was used at a helium gas rate of 0.89mL min -1. The column temperature was held at 60ºC for 1 min, and then increased to 310ºC at a rate of 10ºC/min for 37 minutes. The detector (Agilent 5977A) operated in the electron impact ionization mode (70 eV) and mass spectra were recorded after a solvent delay of 6.5 min. Mass spectrometry operated in SIM mode at scan speed of 1.562u/s and dwell time of 50ms/m/z. A quantifier and a qualifier m/z were monitored for each

of the following targets: pyruvic acid, lactate, succinic acid, fumaric acid, oxaloacetic acid, malic acid, α-ketoglutaric acid, aconitic acid, citric acid/ isocitric acid, glucose, ribose-5 phosphate, glucose-6 phosphate, fructose 1-6 phosphate and succinic acid D4 (IS). Data were analyzed on Agilent MassHunter Quantitative Analysis and normalized by total of protein. Each sample was analyzed in two technical replicates. Statistical Analysis was comparisons used a Student-t test.

Mass spectrometry analysis of ATP, PCr, NAD+ and NADH

ATP, Phosphocreatine, creatine, NAD+ and NADH were measured according to a previous publication [16]. Briefly, cardiomyocytes at day 30 (1x10^6 cells) were washed with cold PBS and metabolites were extracted with 1mL of Methanol: Water (1:1 vol/vol). Solution were dried overnight in speedvac and resuspended in Mass Spectrometry grade water. Metabolites were analyzed in both positive and negative mode in a target assay using 5500 QTRAP hybrid dual quadrupole linear ion trap mass spectrometer.

Supplemental Table 1. Self-reported race and ethnicity of SHaRe participants[†], grouped according to genotypes.

	**Total**	**SARC+**	**SARC-**	**VUS-IHM+**	**VUS-IHM-**
	**N = 2,096**	**N = 494**	**N = 1,471**	**N = 54**	**N = 77**
American Indian or Alaska Native	1 (0.05%)	0 (0%)	0 (0%)	0 (0%)	1 (1.41%)
Asian	55 (2.86%)	10 (2.17%)	39 (2.90%)	1 (2.04%)	5 (7.04%)
Black	72 (3.74%)	6 (1.30%)	60 (4.46%)	2 (4.08%)	4 (5.63%)
More than One	7 (0.36%)	3 (0.65%)	4 (0.30%)	0 (0%)	0 (0%)
Native Hawaiian, Other Pacific Islander	10 (0.52%)	6 (1.30%)	4 (0.30%)	0 (0%)	0 (0%)
Other or Not Reported	84 (4.36%)	20 (4.35%)	55 (4.09%)	3 (6.12%)	6 (8.45%)
White	1,696 (88.10%)	415 (90.22%)	1,183 (87.96%)	43 (87.76%)	55 (77.46%)

[†] There are no significance differences in the ancestries of SHaRe participants with genotypes VUS-IHM+ and VUS-IHM-, or between Sarc+ and VUS-IHM+, or between Sarc-and VUS-IHM- (p= NS, Fisher's Exact).

Supplemental Table 2. Demographics† and Clinical Status of HCM Patients who Provided LV Tissues

	Gender	Age	Ejection fraction	Tissue type	Tissue location	Phenotype
**Normal Control 1**	Female	42	50%	Non-failing heart	Flash frozen LV	Genotype negative control
**Normal Control 2**	Male	38	60%	Non-failing heart	Flash frozen LV	Genotype negative control
**MYH7 R17C**	Male	11	77%	Myectomy	LV proximal septum	VUS IHM+
**MYH7 A226V**	Male	72	65%	Myectomy	LV proximal septum	VUS IHM+
**MYH7 R403Q**	Female	-	<30%	Failing obtained after transplant	Liquid nitrogen frozen LV	Pathogenic HCM
**MYH7 R719W**	Female	40	<30%	Failing obtained after transplant	Liquid nitrogen frozen myocardium	Pathogenic HCM
**MYH7 R719W**	Male	~38	<30%	Failing obtained after transplant	LV free wall liquid nitrogen storage	Pathogenic HCM
**MYH7 Y883H**	Female	44	70%	Myectomy	LV proximal septum	VUS IHM-
**MYH7 E1039G**	Male	39	70%	Myectomy	LV proximal septum	VUS IHM-
**MYH7 R1606C**	Male	59	70%	Myectomy	LV proximal septum	VUS IHM-

† All LV tissues used in Mant-ATP experiments were obtained from HCM patients (all of White race) with gender, age, ejection fraction, and approximate tissue locations indicated. Cardiac ejection fractions ≥ 50% ≤ 64% was considered normal for these studies.

Supplemental Table 3. Analysis of Myosin Isoform Expression†

	WT	*MYH7*$^{R403Q/+}$	*MYH7*$^{R719W/+}$
***MYH7/MYH6* RNA**	0.95 ± 0.02	0.96 ± 0.01<br>NS	0.95 ± 0.01<br>NS
***MYH7/MYH6* Protein**	0.97 ± 0.02	-	-

† Levels of *MYH7/MYH6* transcripts (obtained by bulk RNA sequencing of cultures) and protein (obtained by mass spectrometry) were assessed in day 30 WT iPSC-CMs obtained from ≥ 3 independent differentiations. The percentage of *MYH7* expression was calculated as *MYH7/(MYH6+MYH7*). Statistical significance is denoted in comparison to WT expression levels.

Supplemental Figure 1.

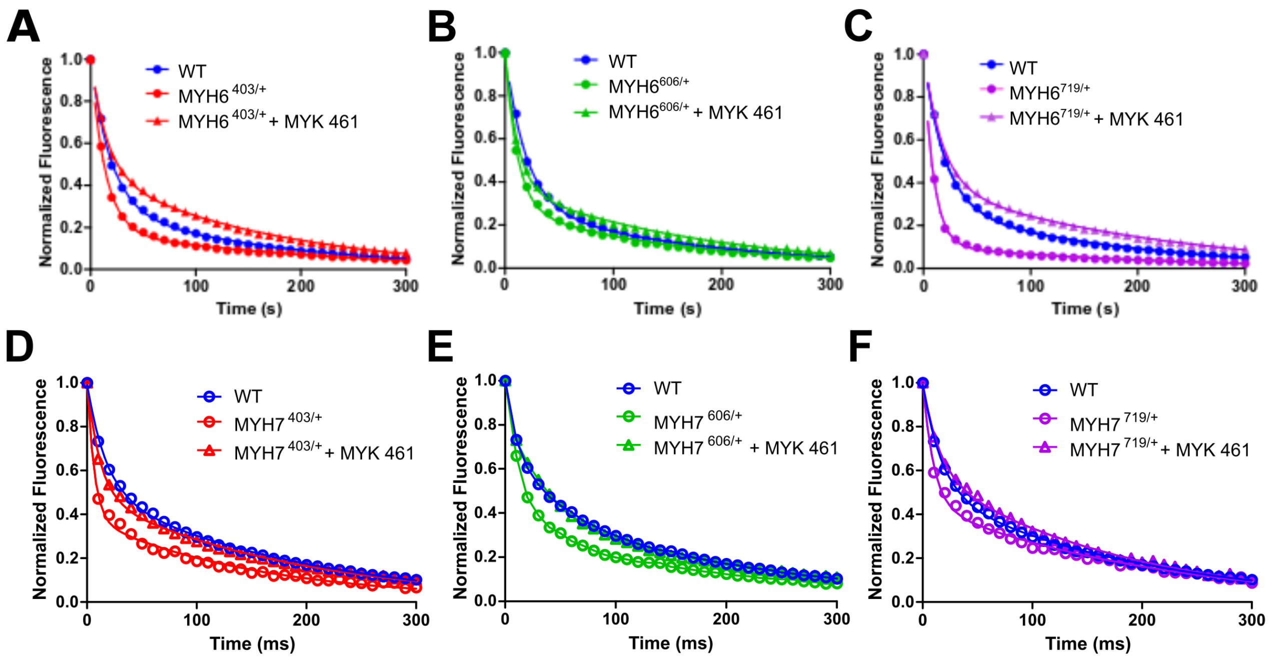

Supplemental Figure 1. Normalized fluorescent decay curves from Mant-ATP experiments. A-C) Averaged fluorescent decay for WT and HCM variants *Myh6*$^{R403Q/+}$, *Myh6*$^{V606M/+}$*, and Myh6*$^{R719W/+}$ in mouse left ventricular myocardium, studied in the presence or absence of 0.3μM MYK-461. Each of the normalized fluorescent decays was fitted with a double exponential decay, from which we derived ratios of DRX and SRX myosin conformations shown in Figure 2 in the main manuscript. **D-F)** Averaged fluorescent decay curves for iPSC-CMs with genotypes WT, *MYH7*$^{R403Q/+}$*, MYH7*$^{V606M/+}$*, and MYH7*$^{R719W/+}$ studied in the presence or absence of 0.3μM MYK-461.

Supplemental Figure 2.

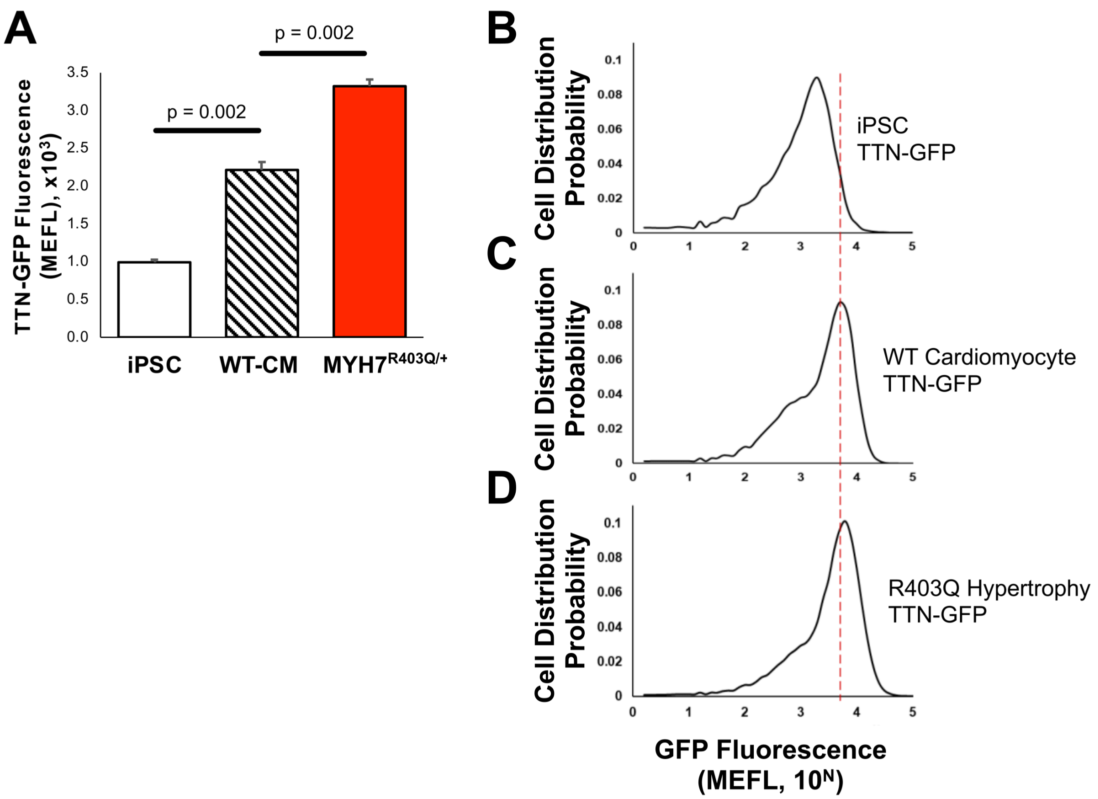

Supplemental Figure 2. The fluorescence of iPSCs and iPSC-CMs expressing titin-tagged with GFP (TTN-GFP), measured by FACS analysis. **A)** GFP intensity measured across three differentiations of iPSC-CMs with genotypes WT and $MYH7^{R403Q}$. **B, C, D)** Representative TTN-GFP fluorescence cell distributions for undifferentiated WT iPSCs (B) and iPSC-CMs (C, WT; D, $MYH7^{R403Q}$). The dotted red highlights the increase in TTN-GFP fluorescence observed with differentiation of iPSCs into cardiomyocytes. Significance was assessed by One-way ANOVA with post-hoc Bonferroni correction for multiple comparisons. Significance cut off of $P < 0.05$ was applied.

Supplemental Figure 3.

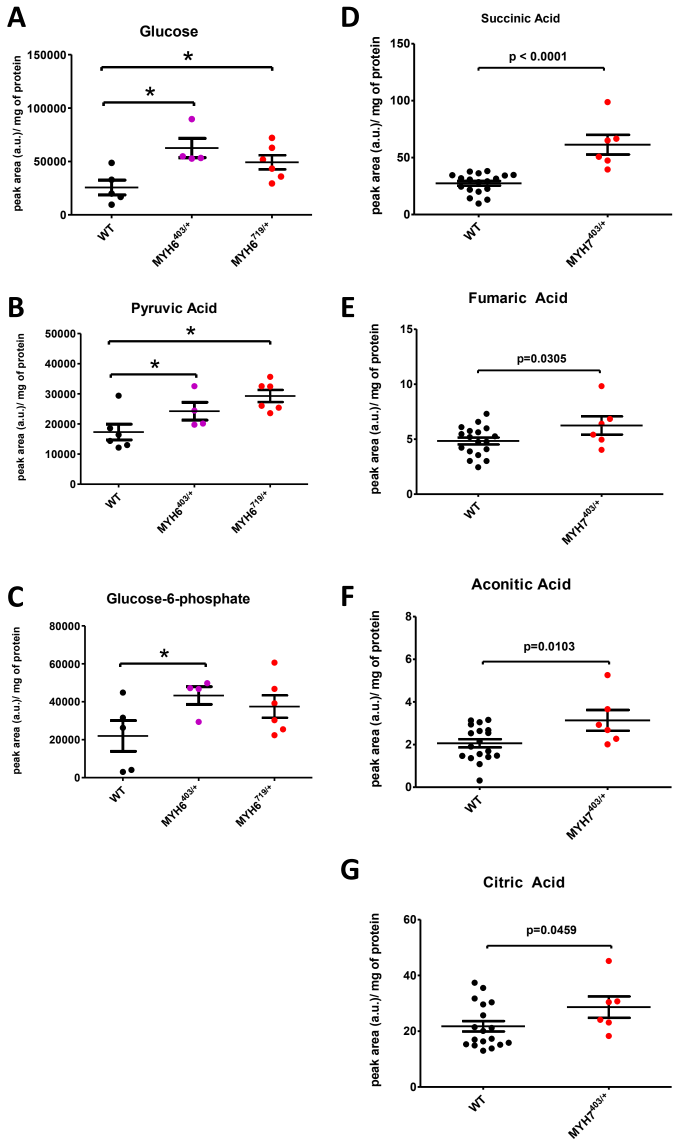

Supplemental Figure 3. Assessment of glycolysis and TCA intermediates in tissues and cells with HCM variants. A-C) Comparison of metabolomics in WT, *Myh6*$^{403/+}$ and *Myh6*$^{719/+}$ mouse ventricular tissues show increased levels of glycolytic metabolites in mutant hearts. Similar increases were found in metabolomics analyses of mutant iPSC-CMs (Figure 4 in manuscript). **D-G)** Comparison of WT and *MYH7*$^{403/+}$ iPSC-CMs demonstrate increased metabolites from the tricarboxylic acid (TCA) cycle in mutant iPSC-CMs, consistent with the increased number of mitochondria and OCR levels observed in Figure 4 in the manuscript. Figure A-C statistical analysis was performed by One-way ANOVA with post-hoc Bonferroni correction for multiple comparisons. Significance cut off of $P < 0.05$ was applied and denoted as *. T-tests defined statistical significance in panels D-G.

Supplemental Figure 4.

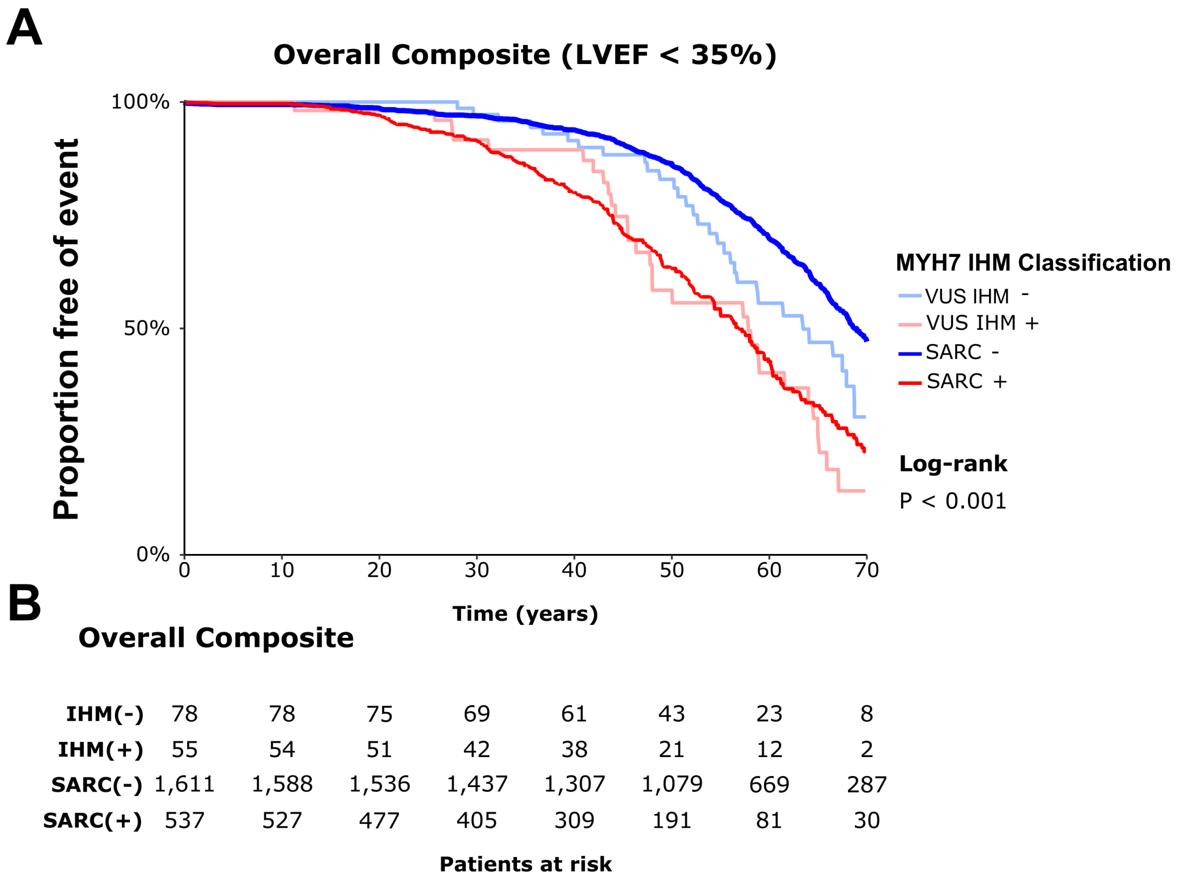

B

Overall Composite

**IHM(-)**	78	78	75	69	61	43	23	8
**IHM(+)**	55	54	51	42	38	21	12	2
**SARC(-)**	1,611	1,588	1,536	1,437	1,307	1,079	669	287
**SARC(+)**	537	527	477	405	309	191	81	30

Patients at risk

Supplemental Figure 4. Composite outcomes in from the SHaRe database. A) Kaplein-Meier curve show relative time points at which SHaRe patients in SARC+, SARC-, VUS-IHM+, and VUS-IHM- groups meet an overall composite endpoint outcome. **B)** The numbers of patients at each time point of sampling (0, 20, 40, 60, 80, 100 years of age) with adverse clinical outcomes for overall composite of all adverse outcomes.

Supplemental References

1. Toepfer CN, Wakimoto H, Garfinkel AC, McDonough B, Liao D, Jiang J, Tai A, Gorham JM, Lunde I, Lun M, et al. Hypertrophic cardiomyopathy mutations in MYBPC3 dysregulate myosin. *Sci Transl Med*. 2019;11; pii: eaat1199. doi: 10.1126/scitranslmed.aat1199.
2. Pasqualin C, Gannier F, Yu A, Malecot CO, Bredeloux P and Maupoil V. SarcOptiM for ImageJ: high-frequency online sarcomere length computing on stimulated cardiomyocytes. *Am J Physiol Cell Physiol*. 2016;311:C277-83.
3. Ho CY, Day SM, Ashley EA, Michels M, Pereira AC, Jacoby D, Cirino AL, Fox JC, Lakdawala NK, Ware JS, et al. Genotype and Lifetime Burden of Disease in Hypertrophic Cardiomyopathy: Insights from the Sarcomeric Human Cardiomyopathy Registry (SHaRe). *Circulation*. 2018;138:1387-1398.
4. McNamara JW, Li A, Smith NJ, Lal S, Graham RM, Kooiker KB, van Dijk SJ, Remedios CGD, Harris SP and Cooke R. Ablation of cardiac myosin binding protein-C disrupts the super-relaxed state of myosin in murine cardiomyocytes. *J Mol Cell Cardiol*. 2016;94:65-71.
5. Hooijman P, Stewart MA and Cooke R. A new state of cardiac myosin with very slow ATP turnover: a potential cardioprotective mechanism in the heart. *Biophys J*. 2011;100:1969-76.
6. Christodoulou DC, Gorham JM, Herman DS and Seidman JG. Construction of normalized RNA-seq libraries for next-generation sequencing using the crab duplex-specific nuclease. *Curr Protoc Mol Biol*. 2011;Chapter 4:Unit4 12.
7. Hinson JT, Chopra A, Nafissi N, Polacheck WJ, Benson CC, Swist S, Gorham J, Yang L, Schafer S, Sheng CC, et al. HEART DISEASE. Titin mutations in iPS cells define sarcomere insufficiency as a cause of dilated cardiomyopathy. *Science*. 2015;349:982-6.
8. Burke MA, Chang S, Wakimoto H, Gorham JM, Conner DA, Christodoulou DC, Parfenov MG, DePalma SR, Eminaga S, Konno T, et al. Molecular profiling of dilated cardiomyopathy that progresses to heart failure. *JCI Insight*. 2016; May 5;1(6). pii: e86898.
9. Navarrete-Perea J, Yu Q, Gygi SP and Paulo JA. Streamlined Tandem Mass Tag (SL-TMT) Protocol: An Efficient Strategy for Quantitative (Phospho)proteome Profiling Using Tandem Mass Tag-Synchronous Precursor Selection-MS3. *J Proteome Res*. 2018;17:2226-2236.
10. Cox J and Mann M. MaxQuant enables high peptide identification rates, individualized p.p.b.-range mass accuracies and proteome-wide protein quantification. *Nat Biotechnol*. 2008;26:1367-72.
11. Schwanhausser B, Busse D, Li N, Dittmar G, Schuchhardt J, Wolf J, Chen W and Selbach M. Global quantification of mammalian gene expression control. *Nature*. 2011;473:337-42.
12. Toepfer CN, Sharma A, Cicconet M, Garfinkel AC, Mucke M, Neyazi M, Willcox JA, Agarwal R, Schmid M, Rao J, et al. SarcTrack: An Adaptable Software Tool for Efficient Large-Scale Analysis of Sarcomere Function in hiPSC-Cardiomyocytes. *Circ Res*. 2019; 124:1172-1183.
13. Padilha K, Venturini G, de Farias Pires T, Horimoto ARVR, Malagrino PA, Gois TC. Kiers B, Oliveira CM, de Oliveira Lavim R, Blatt C, et al. Serum metabolomics profile of type 2 diabetes mellitus in a Brazilian rural population. *Metabolomics*. 2016;15:156.
14. Xia J, Sinelnikov IV, Han B and Wishart DS. MetaboAnalyst 3.0--making metabolomics more meaningful. *Nucleic Acids Res*. 2015;43:W251-7.
15. Calderon-Santiago M, Priego-Capote F, Galache-Osuna JG and Luque de Castro MD. Method based on GC-MS to study the influence of tricarboxylic acid cycle metabolites on cardiovascular risk factors. *J Pharm Biomed Anal*. 2013;74:178-85.
16. Yuan M, Breitkopf SB, Yang X and Asara JM. A positive/negative ion-switching, targeted mass spectrometry-based metabolomics platform for bodily fluids, cells, and fresh and fixed tissue. *Nat Protoc*. 2012;7:872-81.
